# Twenty Years of Neuroinformatics: A Bibliometric Analysis

**DOI:** 10.1007/s12021-024-09712-3

**Published:** 2025-01-15

**Authors:** Miguel Guillén-Pujadas, David Alaminos, Emilio Vizuete-Luciano, José M. Merigó, John D. Van Horn

**Affiliations:** 1 Department of Business, University of Barcelona, Av. Diagonal 690, Barcelona, 08034 Spain; 2 School of Computer Science, Faculty of Engineering and Information Technology, University of Technology Sydney, 81 Broadway, Ultimo, NSW 2007 Australia; 3 Department of Psychology, University of Virginia, Charlottesville, VA 22904 USA; 4 School of Data Science, University of Virginia, Charlottesville, VA 22904 USA

**Keywords:** Bibliometrics, Web of science, Scopus, Keyword analysis, VOS viewer

## Abstract

This study presents a thorough bibliometric analysis of Neuroinformatics over the past 20 years, offering insights into the journal’s evolution at the intersection of neuroscience and computational science. Using advanced tools such as VOS viewer and methodologies like co-citation analysis, bibliographic coupling, and keyword co-occurrence, we examine trends in publication, citation patterns, and the journal’s influence. Our analysis reveals enduring research themes like neuroimaging, data sharing, machine learning, and functional connectivity, which form the core of Neuroinformatics. These themes highlight the journal’s role in addressing key challenges in neuroscience through computational methods. Emerging topics like deep learning, neuron reconstruction, and reproducibility further showcase the journal’s responsiveness to technological advances. We also track the journal’s rising impact, marked by a substantial growth in publications and citations, especially over the last decade. This growth underscores the relevance of computational approaches in neuroscience and the high-quality research the journal attracts. Key bibliometric indicators, such as publication counts, citation analysis, and the *h*-index, spotlight contributions from leading authors, papers, and institutions worldwide, particularly from the USA, China, and Europe. These metrics provide a clear view of the scientific landscape and collaboration patterns driving progress. This analysis not only celebrates Neuroinformatics’s rich history but also offers strategic insights for future research, ensuring the journal remains a leader in innovation and advances both neuroscience and computational science.

## Introduction

Since its inception, Neuroinformatics has established itself as a pivotal peer-reviewed academic journal at the intersection of neuroscience and information science. Published by Springer, the journal serves as a crucial platform for disseminating cutting-edge research, technological advancements, and theoretical discussions within the fields of neuroscience and computational modeling. The journal actively engages a broad readership, including neuroscientists, data scientists, computational modelers, and bioinformaticians, in addition to university faculty, researchers, and students. Covering a wide range of topics such as brain data analysis, neuroimaging techniques, computational models of neural systems, and neuroinformatics tools, the journal’s comprehensive scope is highly regarded. It is indexed in leading databases like PubMed, Scopus, and Web of Science (WoS), which provide detailed insights into its diverse subject matter and thematic clusters.

Throughout its history, Neuroinformatics has been instrumental in fostering communication and collaboration between neuroscience researchers, academics, and computational experts. By providing a robust forum for sharing innovative methodologies, algorithms, and discoveries, the journal has played a key role in advancing neuroinformatics as a discipline. Over the years, it has adapted to the dynamic landscape of neuroscience and technology, incorporating emerging research trends and new computational techniques, further cementing its relevance and impact.

The primary goal of Neuroinformatics is to advance the integration of neuroscience and information science by providing a platform for cutting-edge research in computational modeling, brain data analysis, and neuroinformatics tools. The journal fosters innovation in the development of methods and technologies for analysing complex neural systems, managing large-scale brain data, and simulating neural circuits. Through its interdisciplinary focus, Neuroinformatics contributes significantly to the scientific community by promoting collaboration between neuroscientists, data scientists, and computational modelers, helping to drive progress in understanding the brain’s structure and function. By publishing pioneering research, the journal accelerates discoveries in neuroscience, supports the development of Neuroinformatics as a discipline, and bridges the gap between experimental data and computational insights.

Neuroinformatics continues to make a significant impact on the field, as evidenced by its consistent citation metrics and standing within scientific rankings. According to data from the Journal Citation Reports (JCR), the journal’s impact factor has seen steady fluctuations over the years, reflecting its continued influence in driving research and technological development in Neuroinformatics. Despite these fluctuations, the journal has upheld its stature as a central hub for Neuroinformatics research and remains a vital resource for both scholars and practitioners.

Neuroinformatics has maintained a significant yet fluctuating position within both the Computer Science, Interdisciplinary Applications and Neurosciences categories. In 2023, it ranked 76th out of 169 journals in Computer Science (Q2), and 158th out of 310 in Neurosciences (Q3). Historically, it has performed strongly, particularly in the mid-2000s, achieving Q1 rankings in both fields, such as in 2007, when it reached a top percentile rank of 2nd out of 92 in Neurosciences. The journal is indexed in the Science Citation Index Expanded (SCIE), where it has also seen variability, with a peak in 2007. Despite recent fluctuations, Neuroinformatics remains an influential journal in advancing research at the intersection of neuroscience and computational science.

Anniversaries of a journal have often included special issues (Monastersky & Van Noorden, 2019), editorials (Barley, 2016), and bibliometric assessments to celebrate its achievements and provide a reflective analysis of its impact over time (Cancino et al., 2017; Cobo et al., 2015). These retrospectives offer valuable insights into the journal’s development, citation patterns, and evolving research contributions within the neuroscience and computational science communities (Biemans et al., 2007; Kumar et al., 2023). This paper aims to accomplish two key objectives: first, to provide an overview of the most highly cited papers in Neuroinformatics and second, to identify and analyse trends within the field through rigorous data analysis. A bibliometric approach will be used to extract relevant data and uncover predominant themes in the journal’s publications (Pritchard, 1969), offering a comprehensive understanding of its contributions to advancing neuroscience and computational techniques (Almeida & Vieira, 2023; Blanco-Mesa et al., 2017; Vizuete-Luciano et al., 2023a).

The article is structured to provide a deep analysis of Neuroinformatics’ bibliometric landscape, with sections dedicated to the methods employed, the results derived, and a thematic analysis of key topics and developments. Through this structured approach, the paper aims to consolidate insights and further highlight the journal’s enduring impact on the evolution of Neuroinformatics.

The rest of the paper is structured as follows. Section 2 provides a brief review of the bibliometric methodology used in this analysis. Section 3 outlines the results, focusing on the publication and citation structure, the most cited papers, the most cited documents in journal articles, as well as the leading authors, institutions, and countries/territories contributing to the journal, and the citing articles. Section 4 develops a graphical representation of the bibliographic data for Neuroinformatics using the VOS viewer tool. Section 5 highlights the main conclusions and wraps up the paper.

## Methodology

This article uses bibliometric analysis to collect quantitative data from various indexed sources (Donthu et al., 2021), primarily focusing on academic articles from the WoS Core Collection database (Merigó et al., 2015). WoS was chosen for this study because it indexes Neuroinformatics from 2003, covering all published documents. While WoS is the main source, alternative databases like Scopus and Google Scholar (Bakkalbasi et al., 2006; Bar-Ilan, 2008; Adriaanse & Rensleigh, 2013) could also have been used. In some instances, the Scopus database is included for additional results, through the SciVal platform (Tables 16 and 17).

**Fig. 1:**
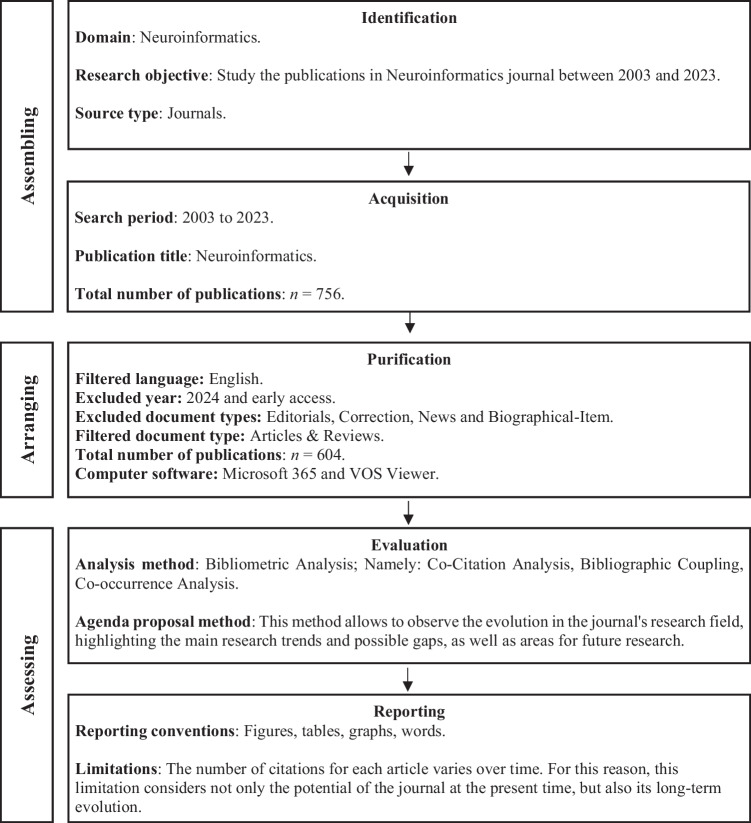
Procedure of the study based on the SPAR-4-SLR protocol

This study’s sample follows clearly defined parameters, relying solely on WoS to guarantee full access to all relevant attributes with high reliability. The bibliometric analysis, as outlined in Figure 1, was conducted using the SPAR-4-SLR Protocol, which includes steps such as identifying the research field, gathering pertinent data, applying specific search filters, and evaluating the results. These procedures are thoroughly explained within the study (Paul et al., 2021; Vizuete-Luciano et al., 2023b).

This work specifically focuses on documents published in Neuroinformatics, narrowing the sample to include only articles and reviews. Different methodologies for conducting bibliometric analysis have been proposed by various researchers (Garfield, 1972; Broadus, 1987; Liao et al., 2019). While some advocate for quantitative literature analysis, others prefer scientific mapping, which has gained popularity recently (Ding et al., 2014; Glanzel et al., 2019). By combining these approaches, the analysis gains greater precision and rigor (Van Noorden et al., 2014; Hicks et al., 2015; Waltman, 2016). 

The study utilizes several performance indicators, such as the number of publications and citations (Garfield, 1955; Elsevier, 2019; Merigó et al., 2018). Although databases provide additional metrics like the h-index to measure citation impact, the g-index and hg-index were not considered in this analysis (Alonso et al., 2009; Okagbue and Teixeira-da Silva, 2020). The focus is on assessing the influence of authors, journals, and universities, incorporating university rankings from the Academic Ranking of World Universities (ARWU) and the Quacquarelli Symonds World University Rankings (QS) (Gaviria-Marín et al., 2018). 

To examine the bibliographic structure and research topics, the study conducts scientific mapping, revealing numerous connections within the scientific landscape (Alaminos et al., 2024; Vizuete-Luciano et al., 2023a). The VOS viewer tool is used to facilitate this mapping (Cobo et al., 2011; Van Eck and Waltman, 2010). VOS viewer employs several bibliometric techniques, such as co-citation analysis (Small, 1973), bibliographic coupling (Kessler, 1963), and the co-occurrence of author keywords (Guan et al., 2024).

## Results

This section presents the findings from a bibliometric analysis of Neuroinformatics over the past two decades. By employing various bibliometric tools and methods, we explored publication trends, citation patterns, co-citation networks, bibliographic coupling, and keyword cooccurrence. The analysis reveals the evolution of research themes, highlights influential contributions, and assesses the journal’s impact within the fields of neuroscience and computational science.

### Publication and Citation Structure of Neuroinformatics

Neuroinformatics began publishing articles in the early 2000s, starting in 2003 with 18 articles. By 2004, the number of publications had increased to 23, but it dropped slightly to 14 in 2005. The number of articles remained relatively stable through the late 2000s, fluctuating between 15 and 20 articles per year. In 2011 and 2012, the journal published 24 and 25 articles, respectively, signalling steady growth. This trend continued into the mid-2010s, with 32 articles published in 2013 and 34 in 2014, maintaining a rate above 30 through 2016. 

**Fig. 2:**
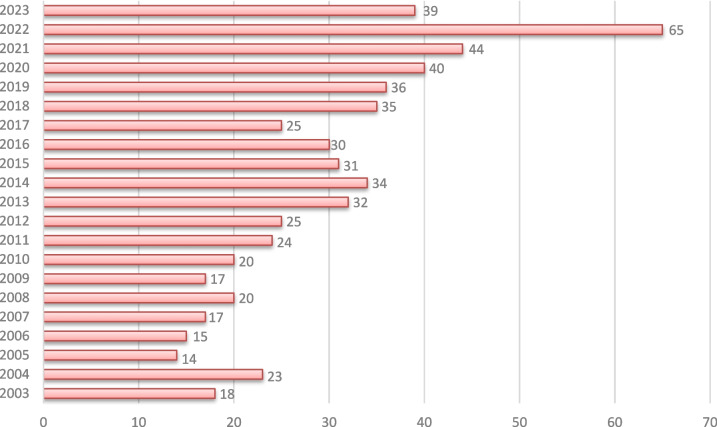
Annual number of papers published in Neuroinformatics

A significant shift occurred in the late 2010s, marked by an increase in the number of published articles, reaching 36 in 2019 and peaking at 44 in 2020. The most notable rise came in 2022, when the journal published a record 65 articles, the highest in its history. This surge in publications reflects a growing interest and advancements in the field of Neuroinformatics, particularly in recent years. The substantial increase in 2023 suggests a surge in research activity and contributions to computational neuroscience, as shown in Figure 2, which illustrates the journal's publication history. This evolution highlights both stability and significant growth in the number of articles published, especially in the early 2020s.

Table 1 outlines the annual citation structure of Neuroinformatics spanning from 2003 to 2023. In 2003, 18 papers accumulated a total of 823 citations, with 2 papers surpassing 100 citations and 5 papers exceeding 50 citations. By 2004, total citations surged to 2,131, with 3 papers achieving over 100 citations and 8 surpassing 50 citations. The data reflect fluctuations in both publication numbers and citation counts, showing significant peaks and troughs. For example, in 2006, despite 15 papers being published, total citations dropped to 269, with no papers exceeding 50 citations, while 2016 recorded an exceptional total of 2,976 citations from 30 papers. 

From 2017 onwards, the number of highly cited papers (≥100 citations) decreased, with most years showing none. Throughout the late 2010s, citation numbers remained moderate, such as 1,139 citations from 35 papers in 2018 and 623 citations from 36 papers in 2019. This trend of lower citation counts continued into the early 2020s, with 2021 and 2022 recording only 510 and 471 citations, respectively, despite higher publication numbers. The decline in citations during recent years is expected, as papers published in later years often require more time to accumulate significant citations.

Over the examined period, Neuroinformatics published 604 papers, amassing a total of 20,132 citations. Among these, 16 papers received 200 or more citations (2.65%), 36 articles obtained 100 or more citations (5.96%), and 589 documents received at least one citation (97.52%). 

**Table 1:** Annual citation structure of Neuroinformatics

Year	TP	TC	TC/TP	≥ 200	≥ 100	≥ 50	≥ 20	≥ 10	≥ 5	≥ 1	T50
2003	18	823	45.72	1	2	5	9	12	15	18	4
2004	23	2,131	92.65	2	4	8	16	19	21	23	6
2005	14	1,244	88.86	1	4	7	12	13	13	14	5
2006	15	269	17.93	0	0	0	7	11	13	15	0
2007	17	933	54.88	2	2	4	9	12	15	17	3
2008	20	610	30.50	0	1	4	9	13	17	20	3
2009	17	1,018	59.88	2	3	3	11	13	16	17	3
2010	20	567	28.35	0	1	4	11	13	18	20	2
2011	24	1,510	62.92	1	5	10	15	18	20	24	6
2012	25	698	27.92	0	0	4	13	18	20	25	1
2013	32	1,283	40.09	2	3	7	10	21	28	32	4
2014	34	1,120	32.94	2	3	3	10	21	29	34	3
2015	31	1,180	38.06	0	4	7	15	24	30	31	4
2016	30	2,976	99.20	1	1	4	13	22	25	28	1
2017	25	502	20.08	0	0	2	9	16	21	25	1
2018	35	1,139	32.54	2	2	4	13	21	29	34	3
2019	36	623	17.31	0	0	2	12	21	30	35	0
2020	40	424	10.60	0	1	1	2	16	28	39	1
2021	44	510	11.59	0	0	0	8	20	28	44	0
2022	65	471	7.25	0	0	1	6	16	30	64	0
2023	39	101	2.59	0	0	0	1	3	6	30	0
Total	604	20,132	821.87	16	36	80	211	343	452	589	50
%	100%	100%		2.65%	5.96%	13.25%	34.93%	56.79%	74.83%	97.52%	8.28%

*TP and TC* = Total papers and citations; ≥200, ≥100, ≥50, ≥20, ≥10, ≥5, ≥1 =Number of papers with equal or more than 200, 100, 50, 20, 10, 5 and 1 citations; *T50* = Number of papers in the Top 50 of Table 3

**Fig. 3:**
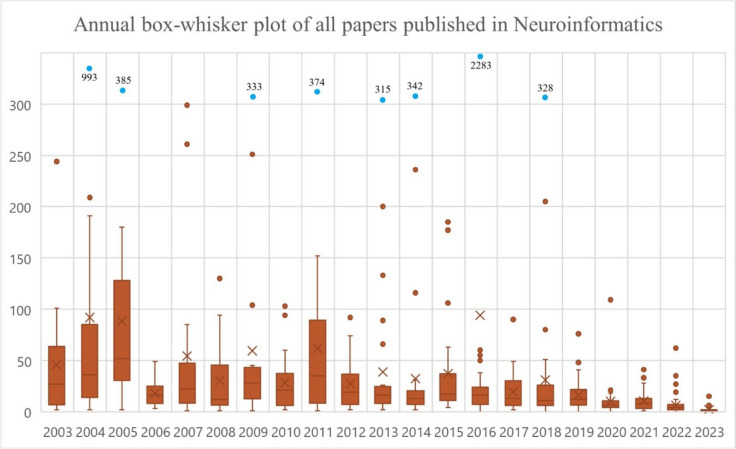
Annual box-plot structure of the citations of all papers published in Neuroinformatics

The box-whisker plot in Figure 3 illustrates the annual citation distribution of all papers published in Neuroinformatics from 2003 to 2023. The plot provides insight into the variability and median citations per year, with outliers represented as individual points (Tukey, 1977). The years 2003 and 2005 show relatively high citation variability, as indicated by the wide range of whiskers and several outliers. In 2016, a notable outlier citation spike is visible, with 2,283 citations for a specific paper, marking it as an exceptional year in terms of research impact. 

Over the years, the median citation rate (depicted by the central line in each box) generally appears stable, though there is a visible decline in the overall citation range starting in the 2010s. This suggests that while some papers continued to receive a high number of citations, most publications received more moderate or fewer citations in the recent decade. The blue dots above the plot represent significant outliers, with values such as 993 in 2003, 385 in 2004, and 328 in 2018. These points reflect particularly influential papers that garnered much higher citations than the yearly median. The decreasing trend in outliers over time suggests that highly cited papers have become less frequent in recent years, aligning with a potential shift in the field’s dynamics or publication and citation patterns. Overall, the plot highlights both the long-term trends in citation impact and notable outliers within Neuroinformatics.

Next, the analysis examines data from the Journal Citation Reports (JCR) within the WoS for Neuroinformatics from 2003 to 2023 (Table 2), focusing on its performance in the Computer Science, Interdisciplinary Applications and Neurosciences categories (Clarivate, 2024). Over this time, total citations (TC) have shown steady growth, peaking at 2,454 citations in 2022. The journal's impact factor (IF) has fluctuated, reaching a high of 5.12 in 2018, while the 5-year impact factor (5YIF), which has been available since 2007, hit its maximum at 5.94 in 2020, reflecting strong long-term relevance. 

The immediacy index (ImIn), which tracks how quickly articles are cited after publication, saw its highest value of 3.95 in 2011, demonstrating that articles from that year were cited rapidly. The number of citable items (CI) has remained relatively consistent, with 68 items in 2020 marking the highest count. The article influence score (AIS), which measures the average impact of articles in the five years after publication, peaked in 2007 at 1.92, though it has varied in recent years, registering at 1.16 in 2023.

In the Computer Science, Interdisciplinary Applications category, Neuroinformatics reached its highest percentile ranking in 2007, at 98.37, consistently ranking in Q1 from 2005 to 2018, signalling its leading position in the field. In the Neurosciences category, the journal achieved a peak percentile of 75.25 in 2005, but has generally been ranked in Q2 or Q3 in more recent years. This review highlights the journal's evolving impact, with notable periods of influence, particularly during the late 2000s and early 2010s.

**Table 2:** Analysis of Neuroinformatics in the JCR of the WoS

Year	TC	IF	5YIF	ImIn	CI	AIS	AJIF	RCSA	Q	PCSA	RNE	Q	PNE
2003	41	-	-	1.72	18	-	-	-	-	-	198/198	Q4	-
2004	64	3.00	-	0.31	19	-	68.43	-	-	-	63/198	Q2	68.43
2005	171	3.90	-	0.57	14	-	85.51	4/83	Q1	95.78	50/200	Q1	75.25
2006	237	3.54	-	1.10	10	-	83.86	4/87	Q1	95.98	57/200	Q2	71.75
2007	301	3.75	4.46	0.52	17	1.92	86.74	2/92	Q1	98.37	53/211	Q2	75.12
2008	348	2.88	3.92	1.00	20	1.27	75.38	7/94	Q1	93.09	94/221	Q2	57.69
2009	474	3.05	4.71	0.58	17	1.43	74.51	8/95	Q1	92.11	100/231	Q2	56.93
2010	470	3.02	3.92	0.50	20	1.26	73.54	9/97	Q1	91.24	106/239	Q2	55.86
2011	568	2.97	2.56	3.95	24	1.08	70.63	13/99	Q1	87.37	113/244	Q2	53.89
2012	653	3.13	3.58	0.40	25	1.29	72.32	12/100	Q1	88.5	111/252	Q2	56.15
2013	619	3.10	2.97	0.46	32	1.19	70.85	12/102	Q1	88.73	119/252	Q2	52.98
2014	786	2.82	3.37	0.44	34	1.06	69.36	13/102	Q1	87.75	124/252	Q2	50.99
2015	899	2.86	3.31	0.90	31	1.34	69.29	15/104	Q1	86.06	122/256	Q2	52.54
2016	1,043	3.20	3.81	0.53	30	1.88	71.19	17/105	Q1	84.29	109/259	Q2	58.11
2017	1,104	3.85	3.46	0.60	25	1.34	79.00	13/105	Q1	88.1	79/261	Q2	69.92
2018	1,277	5.12	4.63	0.57	35	1.45	87.09	9/106	Q1	91.98	48/267	Q1	82.21
2019	1,457	3.30	5.00	1.08	36	1.62	61.29	39/109	Q2	64.68	115/272	Q2	57.9
2020	1,780	4.08	5.94	0.85	68	1.79	63.23	38/111	Q2	66.22	109/273	Q2	60.26
2021	2,155	2.86	5.82	0.72	54	1.59	33.31	67/112	Q3	40.63	204/275	Q3	26
2022	2,454	3.0	4.0	1.0	53	1.26	40.9	65/110	Q3	41.4	163/272	Q3	40.3
2023	2,099	2.7	3.3	0.7	25	1.16	52.3	76/169	Q2	55.3	158/310	Q3	49.2

*TC *= Total citations; *IF* = Impact factor; *5YIF* = 5-year impact factor; *ImIn* = Immediacy index; *CI *= Citable items; *AIS* = Article Influence Score; *AJIF* = Average journal impact factor percentile; *RCSA* = Ranking in the WoS category of Computer Science, Interdisciplinary Applications; *Q *= Quartile in CSA; *PCSA* = Journal impact factor percentile in Computer Science, Interdisciplinary Applications; *RNE* = Ranking in the WoS category of Neurosciences; *Q* = Quartile in NE; *PNE* = Journal impact factor percentile in Neurosciences

### Influential Papers in Neuroinformatics

As highlighted in Table 3, the 50 most cited documents in Neuroinformatics provide a clear view of the journal's most impactful contributions to the field. The top-cited paper, "DPABI: Data Processing & Analysis for (Resting-State) Brain Imaging" by Yan et al. (2016), stands out with 2,283 citations, averaging an impressive 285.38 citations per year, reflecting its central role in advancing brain imaging research. Similarly, "The Small World of the Cerebral Cortex" by Sporns and Zwi (2004) has accrued 993 citations, underscoring the continued relevance of theoretical research on brain network structures. 

Other highly cited works include "BrainMap - The Social Evolution of a Human Brain Mapping Database" by Laird et al. (2005) with 385 citations, highlighting the critical importance of data-sharing platforms in the neuroimaging community. "An Open Source Multivariate Framework for n-Tissue Segmentation with Evaluation on Public Data" by Avants et al. (2011), with 374 citations, and "PyMVPA: A Python Toolbox for Multivariate Pattern Analysis of fMRI Data" by Hanke et al. (2009), with 333 citations, demonstrate the growing influence of opensource tools and multivariate pattern analysis in the field. 

Papers such as "SegAN: Adversarial Network with Multi-scale L1 Loss for Medical Image Segmentation" by Xue et al. (2018), cited 328 times, highlight the integration of machine learning techniques, specifically adversarial networks, into medical image analysis. The prominence of neuroimaging toolkits like "PRoNTo: Pattern Recognition for Neuroimaging Toolbox" by Schrouff et al. (2013) and "The Extensible Neuroimaging Archive Toolkit" by Marcus et al. (2007), each with over 300 citations, reinforces the field’s reliance on sophisticated software platforms.

**Table 3:** The 50 most cited documents of Neuroinformatics

*R*	TC	Title	Author/s	Year	C/Y
1	2,283	DPABI: Data Processing & Analysis for (Resting-State) Brain Imaging	Yan, CG; Wang, XD; Zuo, XN; Zang, YF	2016	285.38
2	993	The small world of the cerebral cortex	Sporns, O; Zwi, JD	2004	49.65
3	385	BrainMap - The social evolution of a human brain mapping database	Laird, AR; Lancaster, JL; Fox, PT	2005	20.26
4	374	An Open Source Multivariate Framework for n-Tissue Segmentation with Evaluation on Public Data	Avants, BB; Tustison, NJ; Wu, J; Cook, PA; Gee, JC	2011	28.77
5	342	A Review of Feature Reduction Techniques in Neuroimaging	Mwangi, B; Tian, TS; Soares, JC	2014	34.20
6	333	PyMVPA: a Python Toolbox for Multivariate Pattern Analysis of fMRI Data	Hanke, M; Halchenko, YO; Sederberg, PB; Hanson, SJ; Haxby, JV; Pollmann, S	2009	22.20
7	328	SegAN: Adversarial Network with Multi-scale L_1_ Loss for Medical Image Segmentation	Xue, Y; Xu, T; Zhang, H; Long, LR; Huang, X	2018	54.67
8	315	PRoNTo: Pattern Recognition for Neuroimaging Toolbox	Schrouff, J; Rosa, MJ; Rondina, JM; Marquand, AF; Chu, C; Ashburner, J; Phillips, C; Richiardi, J; Mourao-Miranda, J	2013	28.64
9	299	The Extensible Neuroimaging Archive Toolkit: An informatics platform for managing, exploring, and sharing neuroimaging data	Marcus, DS; Olsen, TR; Ramaratnam, M; Buckner, RL	2007	17.59
10	261	Web-based method for translating neurodevelopment from laboratory species to humans	Clancy, B; Kersh, B; Hyde, J; Darlington, RB; Anand, KJS; Finlay, BL	2007	15.35
11	251	Visualization of Group Inference Data in Functional Neuroimaging	Glaescher, J	2009	16.73
12	244	WebQTL - Web-based complex trait analysis	Wang, JT; Williams, RW; Manly, KF	2003	11.62
13	236	A Standardized [< SUP > 18</SUP > F]-FDG-PET Template for Spatial Normalization in Statistical Parametric Mapping of Dementia	Della Rosa, PA; Cerami, C; Gallivanone, F; Prestia, A; Caroli, A; Castiglioni, I; Gilardi, MC; Frisoni, G; Friston, K; Ashburner, J; Perani, D	2014	23.60
14	209	Online retrieval, processing, and visualization of primate connSectivity data from the CoCoMac database	Kötter, R	2004	10.45
15	205	Multi-Modality Cascaded Convolutional Neural Networks for Alzheimer’s Disease Diagnosis	Liu, M; Cheng, D; Wang, K; Wang, Y	2018	34.17
16	200	HERMES: Towards an Integrated Toolbox to Characterize Functional and Effective Brain Connectivity	Niso, G; Bruna, R; Pereda, E; Gutierrez, R; Bajo, R; Maestu, F; del-Pozo, F	2013	18.18
17	191	Clustered organization of cortical connectivity	Hilgetag, CC; Kaiser, M	2004	9.55
18	185	The Scalable Brain Atlas: Instant Web-Based Access to Public Brain Atlases and Related Content	Bakker, R; Tiesinga, P; Kotter, R	2015	20.56
19	180	Statistical criteria in fMRI studies of multisensory integration	Beauchamp, MS	2005	9.47
20	177	Optimal Symmetric Multimodal Templates and Concatenated Random Forests for Supervised Brain Tumor Segmentation (Simplified) with ANTsR	Tustison, NJ; Shrinidhi, KL; Wintermark, M; Durst, CR; Kandel, BM; Gee, JC; Grossman, MC; Avants, BB	2015	19.67
21	152	A Broadly Applicable 3-D Neuron Tracing Method Based on Open-Curve Snake	Wang, Y; Narayanaswamy, A; Tsai, CL; Roysam, B	2011	11.69
22	149	Unified Framework for Development, Deployment and Robust Testing of Neuroimaging Algorithms	Joshi, A; Scheinost, D; Okuda, H; Belhachemi, D; Murphy, I; Staib, LH; Papademetris, X	2011	11.46
23	133	The MCIC Collection: A Shared Repository of Multi-Modal, Multi-Site Brain Image Data from a Clinical Investigation of Schizophrenia	Gollub, RL; Shoemaker, JM; King, MD; White, T; Ehrlich, S; Sponheim, SR; Clark, VP; Turner, JA; Mueller, BA; Magnotta, V; O’Leary, D; Ho, BC; Brauns, S; Manoach, DS; Seidman, L; Bustillo, JR; Lauriello, J; Bockholt, J; Lim, KO; Rosen, BR; Schulz, SC; Calhoun, VD; Andreasen, NC	2013	12.09
24	131	Simulation and robotics studies of salamander locomotion - Applying neurobiological principles to the control of locomotion in robots	Ijspeert, AJ; Crespi, A; Cabelguen, JM	2005	6.89
25	130	The Neuroscience Information Framework: A Data and Knowledge Environment for Neuroscience	Gardner, D; Akil, H; Ascoli, GA; Bowden, DM; Bug, W; Donohue, DE; Goldberg, DH; Grafstein, B; Grethe, JS; Gupta, A; Halavi, M; Kennedy, DN; Marenco, L; Martone, ME; Miller, PL; Mueller, HM; Robert, A; Shepherd, GM; Sternberg, PW; Van Essen, DC; Williams, RW	2008	8.13
26	127	Phase synchronization measurements using electroencephalographic recordings - What can we really say about neuronal synchrony?	Guevara, R; Velazquez, JLP; Nenadovic, V; Wennberg, R; Senjanovic, G; Dominguez, LG	2005	6.68
27	116	Validation of FreeSurfer-Estimated Brain Cortical Thickness: Comparison with Histologic Measurements	Cardinale, F; Chinnici, G; Bramerio, M; Mai, R; Sartori, I; Cossu, M; Lo Russo, G; Castana, L; Colombo, N; Caborni, C; De Momi, E; Ferrigno, Go	2014	11.60
28	113	Contexts and catalysts - A resolution of the localization and integration of function in the brain	McIntosh, AR	2004	5.65
29	109	3D-Deep Learning Based Automatic Diagnosis of Alzheimer’s Disease with Joint MMSE Prediction Using Resting-State fMRI	Nguyen TD; Ryu, S; Qureshi, MNI; Choi, M; Lee, KH; Lee, B	2020	27.25
30	108	Clinical Prediction from Structural Brain MRI Scans: A Large-Scale Empirical Study	Sabuncu, MR; Konukoglu, E	2015	12.00
31	106	Automatic Detection of White Matter Hyperintensities in Healthy Aging and Pathology Using Magnetic Resonance Imaging: A Review	Caligiuri, ME; Perrotta, P; Augimeri, A; Rocca, F; Quattrone, A; Cherubini, A	2015	11.78
32	104	NETMORPH: A Framework for the Stochastic Generation of Large Scale Neuronal Networks With Realistic Neuron Morphologies	Koene, RA; Tijms, B; van Hees, P; Postma, F; de Ridder, A; Ramakers, GJA; van Pelt, J; van Ooyen, A	2009	6.93
33	103	The DIADEM Data Sets: Representative Light Microscopy Images of Neuronal Morphology to Advance Automation of Digital Reconstructions	Brown, KM; Barrionuevo, G; Canty, AJ; De Paola, V; Hirsch, JA; Jefferis, GSXE; Lu, J; Snippe, M; Sugihara, I; Ascoli, GA	2011	7.92
34	103	Automated Reconstruction of Dendritic and Axonal Trees by Global Optimization with Geometric Priors	Tueretken, E; Gonzalez, G; Blum, C; Fua, P	2011	7.92
35	103	The Java Image Science Toolkit (JIST) for Rapid Prototyping and Publishing of Neuroimaging Software	Lucas, BC; Bogovic, JA; Carass, A; Bazin, PL; Prince, JL; Pham, DL; Landman, BA	2010	7.36
36	101	Genetic correlates of gene expression in recombinant inbred strains - A relational model system to explore neurobehavioral phenotypes	Chesler, EJ; Wang, JT; Lu, L; Qu, YH; Manly, KF; Williams, RW	2003	4.81
37	94	Automated Reconstruction of Neuronal Morphology Based on Local Geometrical and Global Structural Models	Zhao, T; Xie, J; Amat, F; Clack, N; Ahammad, P; Peng, H; Long, F; Myers, E	2011	7.23
38	94	Removal of Muscle Artifacts from EEG Recordings of Spoken Language Production	Vos, DM; Ries, S; Vanderperren, K; Vanrumste, B; Alario, FX; Huffel, VS; Burle, B	2010	6.71
39	94	The NIFSTD and BIRNLex Vocabularies: Building Comprehensive Ontologies for Neuroscience	Bug, WJ; Ascoli, GA; Grethe, JS; Gupta, A; Fennema-Notestine, C; Laird, AR; Larson, SD; Rubin, D; Shepherd, GM; Turner, JA; Martone, ME	2008	5.88
40	92	The Cognitive Paradigm Ontology: Design and Application	Turner, JA; Laird, AR	2012	7.67
41	92	Connectivity and dynamics of neural information processing	Jirsa, VK	2004	4.60
42	90	Multimodal Neuroimaging in Schizophrenia: Description and Dissemination	Aine, CJ; Bockholt, HJ; Bustillo, JR; Canive, JM; Caprihan, A; Gasparovic, C; Hanlon, FM; Houck, JM; Jung, RE; Lauriello, J; Liu, J; Mayer, AR; Perrone-Bizzozero, NI; Posse, S; Stephen, JM; Turner, JA; Clark, VP; Calhoun, VD	2017	12.86
43	90	EMAP and EMAGE - A framework for understanding spatially organized data	Baldock, RA; Bard, JBL; Burger, A; Burton, N; Christiansen, J; Feng, GJ; Hill, B; Houghton, D; Kaufman, M; Rao, JG; Sharpe, J; Ross, A; Stevenson, P; Venkataraman, S; Waterhouse, A; Yang, Y; Davidson, DR	2003	4.29
44	89	Obscuring Surface Anatomy in Volumetric Imaging Data	Milchenko, M; Marcus, D	2013	8.09
45	87	The cell-centered database - A database for multiscale structural and protein localization data from light and electron microscopy	Martone, ME; Zhang, SL; Gupta, A; Qian, XF; He, HY; Price, DL; Wong, M; Santini, S; Ellisman, MH	2003	4.14
46	85	Feasibility of multi-site clinical structural neuroimaging studies of aging using legacy data	Fennema-Notestine, C; Gamst, AC; Quinn, BT; Pacheco, J; Jernigan, TL; Thal, L; Buckner, R; Killiany, R; Blacker, D; Dale, AM; Fischl, B; Dickerson, B; Gollub, RL	2007	5.00
47	85	Dynamic connectivity in neural systems - Theoretical and empirical considerations	Breakspear, M	2004	4.25
48	84	Methods for quantifying the informational structure of sensory and motor data	Lungarella, M; Pegors, T; Bulwinkle, D; Sporns, O	2005	4.42
49	81	Data sharing for computational neuroscience	Teeters, JL; Harris, KD; Millman, KJ; Olshausen, BA; Sommer, FT	2008	5.06
50	80	A Topological Representation of Branching Neuronal Morphologies	Kanari, L; Dlotko, P; Scolamiero, M; Levi, R; Shillcock, J; Hess, K; Markram, H	2018	13.33

Table 4 highlights the 50 most cited documents published in Neuroinformatics and related journals, showcasing the journal's impact on various fields such as neuroimaging, computational neuroscience, and brain mapping. The most cited paper in the list is Jenkinson et al. (2012), published in Neuroimage, with 32 citations, reflecting the importance of this work in advancing brain imaging techniques. Similarly, Ascoli et al. (2007), published in the Journal of Neuroscience, also ranks highly with 31 citations, demonstrating significant contributions to neuroscience through neural circuit studies. Notably, Tzourio-Mazoyer et al. (2002) and Fischl et al. (2002), both published in Neuroimage, with 31 and 30 citations, respectively, emphasizing their foundational work in neuroimaging and cortical surface-based analysis. 

Papers like Smith et al. (2004), with 30 citations, and Fischl (2012), with 28 citations, further highlight Neuroinformatics' key role in publishing influential neuroimaging studies that push forward the development of advanced brain mapping techniques. Several other papers from different years continue this trend, such as Avants et al. (2008) in Medical Image Analysis with 25 citations and Cox (1996) with 24 citations, illustrating the journal’s consistent contributions to computational tools and brain imaging analysis. 

Interestingly, older foundational works like Tibshirani (1996) and Dice (1945), each cited 22 times, show that foundational statistical and methodological concepts in data analysis remain highly relevant to current Neuroinformatics research. Additionally, Wang et al. (2011), published in Neuroinformatics with 21 citations, underscores the journal’s role in advancing algorithmic and computational methods for brain connectivity studies. 

Throughout the list, the dominance of neuroimaging-focused articles is clear, but the inclusion of works such as Meijering (2010) in Cytometry Part A, and Peng (2010) in Nature Biotechnology highlights the interdisciplinary nature of Neuroinformatics, merging biology, machine learning, and image analysis. Contributions to foundational neuroscience research, like Hines and Carnevale (1997) in Neural Computation and Ashburner and Friston (2005) in Neuroimage, also demonstrate how the field of Neuroinformatics continues to bridge gaps between computational advancements and neurological discoveries.

**Table 4:** Top 50 most cited documents in Neuroinformatics publications

Rank	Year	First author	Reference	Vol	Page	Type	TC
1	2012	Jenkinson M	Neuroimage	v62	p782	A	32
2	2007	Ascoli GA	J Neurosci	v27	p9247	A	31
3	2002	Tzourio-Mazoyer N	Neuroimage	v15	p273	A	31
4	2002	Fischl B	Neuron	v33	p341	A	30
5	2004	Smith SM	Neuroimage	v23	ps208	A	30
6	2012	Fischl B	Neuroimage	v62	p774	A	28
7	2002	Smith SM	Hum Brain Mapp	v17	p143	A	28
8	2008	Jack CR	J Magn Reson Imaging	v27	p685	A	26
9	2010	Meijering E	Cytom Part A	v77a	p693	A	26
10	2008	Avants BB	Med Image Anal	v12	p26	A	25
11	1996	Cox RW	Comput Biomed Res	v29	p162	A	24
12	2010	Peng HC	Nat Biotechnol	v28	p348	A	24
13	2008	Scorcioni R	Nat Protoc	v3	p866	A	23
14	1945	Dice LR	Ecology	v26	p297	A	22
15	2009	Klein A	Neuroimage	v46	p786	A	22
16	1998	Sled JG	IEEE T Med Imaging	v17	p87	A	22
17	1996	Tibshirani R	J Roy Stat Soc B	v58	p267	A	22
18	1999	Dale AM	Neuroimage	v9	p179	A	21
19	2011	Wang Y	Neuroinformatics	v9	p193	A	21
20	1997	Hines ML	Neural Comput	v9	p1179	A	20
21	2013	Xiao H	Bioinformatics	v29	p1448	A	20
22	2005	Ashburner J	Neuroimage	v26	p839	A	19
23	1998	Cannon RC	J Neurosci Meth	v84	p49	A	19
24	2002	Jenkinson M	Neuroimage	v17	p825	A	19
25	2016	Klikauer T	Triplec-Commun Capit	v14	p260	A	19
26	2009	Bullmore ET	Nat Rev Neurosci	v10	p186	A	18
27	1979	Otsu N	IEEE T Syst Man Cyb	v9	p62	A	18
28	2006	Yushkevich PA	Neuroimage	v31	p1116	A	18
29	2000	Ashburner J	Neuroimage	v11	p805	A	17
30	1995	Benjamini Y	J R Stat Soc B	v57	p289	A	17
31	2003	Bowden DM	Neuroinformatics	v1	p43	A	17
32	2013	Van Essen DC	Neuroimage	v80	p62	A	17
33	2011	Avants BB	Neuroimage	v54	p2033	A	16
34	2011	Chang CC	ACM T Intel Syst Tec	v2		A	16
35	2006	Desikan RS	Neuroimage	v31	p968	A	16
36	2011	Donohue DE	Brain Res Rev	v67	p94	A	16
37	1991	Felleman DJ	Cereb Cortex	v1	p1	A	16
38	2011	Peng HC	Bioinformatics	v27	pi239	A	16
39	2015	Peng HC	Neuron	v87	p252	A	16
40	2005	Sporns O	PLOS Comput Biol	v1	p245	A	16
41	2008	Gardner D	Neuroinformatics	v6	p149	A	15
42	2016	Gorgolewski KJ	Sci Data	v3		A	15
43	2013	Parekh R	Neuron	v77	p1017	A	15
44	2015	Ronneberger O	Lect Notes Comput Sc	v9351	p234	A	15
45	2005	Wearne SL	Neuroscience	v136	p661	A	15
46	2001	Zhang YY	IEEE T Med Imaging	v20	p45	A	15
47	2002	Al-Kofahi KA	IEEE T Inf Technol B	v6	p171	A	14
48	2005	Beckmann CF	Philos T R Soc B	v360	p1001	A	14
49	2001	Breiman L	Machine Learning	v45	p5	A	14
50	2011	Brown KM	Neuroinformatics	v9	p143	A	14

*A* Article, *B* Book

### Citing Articles of Neuroinformatics

**Fig. 4:**
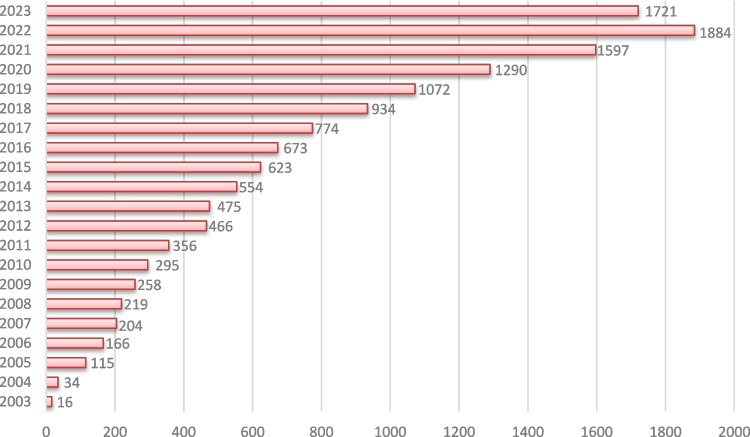
Annual number of citing articles to Neuroinformatics

Figure 4 presents the annual number of citing articles for Neuroinformatics from 2003 to 2023, showcasing a clear upward trend in citation activity over the years. Starting with only 16 citing articles in 2003, the journal has seen a steady increase in citations, reflecting its growing influence and visibility in the scientific community. 

A significant rise is observed from 2013 onwards, with the number of citing articles consistently surpassing 400 annually. By 2019, the journal crossed the 1,000 citing articles mark, reaching 1,072 citing articles that year. This growth continued sharply, peaking in 2022 with 1,884 citing articles, followed closely by 2023, which had 1,721 citing articles. The data for 2020 and 2021 also highlight strong citation activity, with 1,597 and 1,290 citing articles, respectively. 

The early years (2003–2010) show a relatively slow accumulation of citations, with under 300 citing articles per year. However, from 2011 onwards, the journal's impact expanded significantly, as evidenced by the larger number of citations. This pattern indicates that Neuroinformatics has established itself as a core resource within the field, with its work increasingly referenced by other researchers, particularly in recent years.

The upward trend in citing articles points to the journal's growing relevance and recognition, highlighting its critical role in advancing computational neuroscience and Neuroinformatics research.

Table 5 provides an analysis of the top universities, countries, and publishers that have cited articles from Neuroinformatics. The data illustrates the global impact and widespread engagement with the journal’s research across leading academic institutions and countries, as well as the major publishing houses involved. 

Harvard University ranks first, with 565 citing articles, showing its leading role in engaging with Neuroinformatics publications. Other top institutions include the Chinese Academy of Sciences with 417 citations, Yale University with 367, and University College London (UCL) with 352 citations. European institutions, like the CNRS France (326) and the Max Planck Society (296), also show significant engagement, underscoring the journal's global academic reach. 

The USA leads by a significant margin, with 5,348 citing articles, reflecting the strong influence of Neuroinformatics in American research institutions. China follows with 3,790 citing articles, indicating the journal's growing importance in Chinese academic research. Other major contributors include Germany (1,630), the UK (1,508), and Canada (881), demonstrating a solid European and North American presence. Notably, countries like Australia (569) and South Korea (374) are also wellrepresented, highlighting the journal's global engagement. 

Among the publishers, Elsevier is the most prominent, with 3,647 citing articles, reflecting its dominance in academic publishing. Springer Nature follows with 1,668 citing articles, while Frontiers Media (1,564) and Wiley (1,157) also play significant roles. Other notable publishers include Oxford University Press (554) and IEEE (537), emphasizing the interdisciplinary nature of the research citing Neuroinformatics.

This data demonstrates that Neuroinformatics is widely cited by leading universities and research centres around the world, particularly in the USA, China, and Europe. The presence of high citation counts from top-tier publishers like Elsevier and Springer Nature further underscores the journal’s broad influence and integration into both academic and practical applications in computational neuroscience. 

**Table 5:** Citing articles of neuroinformatics: universities, countries, and publishers

*R*	University	TP	Country	TP	Publishers	TP
1	Harvard U	565	USA	5,348	Elsevier	3,647
2	Chinese Academy Sci	417	PR China	3,790	Springer Nature	1,668
3	Yale U	367	Germany	1,630	Frontiers Media	1,564
4	U College London	352	UK	1,508	Wiley	1,157
5	CNRS France	326	Canada	881	Oxford U Press	554
6	Helmholtz Association	322	Italy	782	IEEE	537
7	INSERM France	303	Netherlands	730	Public Library Science	511
8	Max Planck Society	296	France	644	MDPI	432
9	Massachusetts General Hospital	286	Australia	569	Nature Portfolio	432
10	U Pennsylvania	280	Spain	554	Humana Press Inc	285
11	U California San Diego	245	Switzerland	527	IOP Publishing Ltd	190
12	U Toronto	243	South Korea	374	Sage	166
13	McGill U	240	Japan	368	Soc Neuroscience	166
14	Capital Medical U	224	India	284	Taylor & Francis	166
15	Swiss Federal Institutes Tech	223	Belgium	254	Lippincott Williams & Wilkins	142
16	Beijing Normal U	222	Sweden	251	Hindawi Publishing Group	120
17	Johns Hopkins U	213	Brazil	184	MIT Press	120
18	Stanford U	213	Singapore	173	IOS Press	98
19	Zhejiang U	213	Austria	153	Cambridge U Press	82
20	King S College London	200	Norway	152	Mary Ann Liebert, Inc	72
21	U North Carolina Chapel Hill	187	Poland	147	Natl Acad Sciences	71
22	U Oxford	185	Taiwan	147	World Scientific	64
23	U Cambridge	183	Finland	142	Amer Physical Soc	60
24	Research Center Julich	182	Denmark	141	Amer Physiological Soc	45
25	U California Los Angeles	179	Iran	141	Bentham Science Publ Ltd	44
26	Emory U	178	Israel	114	Royal Soc London	43
27	U Chinese Academy Sci	178	Turkey	102	eLife Sciences Publ LTD	38
28	Central South U	174	Greece	96	Karger	36
29	Radboud U Nijmegen	174	Saudi Arabia	93	Dove Medical Press Ltd	34
30	U Southern California	172	Portugal	86	SPIE-Soc Photo-Optic Instr Eng	32

Table 6 provides a breakdown of the journals and research areas that frequently cite articles from Neuroinformatics. This analysis showcases the interdisciplinary reach and the wide variety of fields influenced by research published in the journal. 

Neuroimage is the leading journal citing Neuroinformatics, with 773 citing articles, reflecting the journal's close relationship with neuroimaging research. Other prominent citing journals include Frontiers in Neuroscience (372), PLOS One (358), and Human Brain Mapping (333). These journals are highly regarded in the fields of neuroscience and medical imaging, which aligns with Neuroinformatics’ emphasis on computational methods and neuroimaging. Interestingly, Neuroinformatics itself appears on the list, with 283 self-citations, suggesting that the journal's published research is integral to its ongoing scholarly discourse. The presence of Cerebral Cortex (230citations) and Frontiers in Neuroinformatics (226 citations) further underlines the journal’s influence in core areas of neuroscience and brain mapping. 

In terms of research areas, Neurosciences and Neurology dominate with 7,067 citing articles, underscoring the journal’s central role in advancing knowledge in brain science. Radiology, Nuclear Medicine, and Medical Imaging follows with 1,998 citing articles, highlighting the journal’s importance in imaging-related fields. Other significant research areas include Computer Science (1,823 citing articles), Engineering (1,402 articles), and Psychology (1,011 articles), demonstrating the journal's interdisciplinary impact beyond neuroscience into technology and behavioural sciences. 

Further down the list, fields like Mathematical and Computational Biology (868 citing articles), Psychiatry (910 articles), and Biochemistry and Molecular Biology (774 articles) show the journal’s influence on both the computational and clinical sides of brain research. 

The data reveals that Neuroinformatics plays a vital role across a broad spectrum of scientific areas, from foundational neuroscience to applied engineering and medical informatics. The strong presence of neuroscience-related journals and research areas, such as Neuroimage and Neurosciences and Neurology, emphasizes the journal’s specialized focus, while its influence in fields like Computer Science and Mathematical Biology highlights the growing relevance of computational methods in brain research. 

This interdisciplinary citation pattern shows that Neuroinformatics is not only central to traditional brain science research but also to fields that leverage computational, engineering, and medical innovations, further reflecting its role as a key driver of technological and methodological advancements in neuroscience. 

**Table 6:** Citing articles of neuroinformatics: journals and Research Area

*R*	Journal	TP	Research Area	TP
1	Neuroimage	773	Neurosciences Neurology	7,067
2	Frontiers in Neuroscience	372	Radiology Nuclear Medicine Medical Imaging	1,998
3	PLOS One	358	Computer Science	1,823
4	Human Brain Mapping	333	Engineering	1,402
5	Scientific Reports	299	Science Technology Other Topics	1,084
6	Neuroinformatics	283	Psychology	1,011
7	Cerebral Cortex	230	Psychiatry	910
8	Frontiers in Neuroinformatics	226	Mathematical Computational Biology	868
9	Frontiers in Human Neuroscience	190	Biochemistry Molecular Biology	774
10	Neuroimage Clinical	188	Behavioral Sciences	427
11	J Neuroscience	140	Physics	351
12	Frontiers in Aging Neuroscience	136	Life Sciences Biomedicine Other Topics	297
13	Frontiers in Neurology	135	Mathematics	274
14	Brain Imaging and Behavior	132	Geriatrics Gerontology	252
15	J Neuroscience Methods	132	Pharmacology Pharmacy	243
16	PLOS Computational Biology	127	Medical Informatics	220
17	Frontiers in Psychiatry	126	Cell Biology	204
18	IEEE Access	110	Biotechnology Applied Microbiology	193
19	J Affective Disorders	107	Anatomy Morphology	175
20	Brain Structure Function	97	Genetics Heredity	168
21	IEEE Transactions on Medical Imaging	96	Chemistry	166
22	Neuroscience	94	Imaging Science Photographic Technology	165
23	Medical Image Analysis	91	Physiology	146
24	J Neural Engineering	90	Telecommunications	130
25	Brain Sciences	79	General Internal Medicine	120
26	Neurocomputing	74	Research Experimental Medicine	116
27	J Alzheimers Disease	73	Surgery	112
28	Proc National Academy Sciences USA	71	Biophysics	93
29	Biomedical Signal Processing Control	68	Developmental Biology	90
30	Computers Biology Medicine	68	Instruments Instrumentation	88

### Leading Authors, Institutions, and Countries

Table 7 showcases the top 35 leading authors contributing to Neuroinformatics, providing insights into the impact and productivity of researchers in the field. The table ranks authors based on several key metrics such as total papers (TP), total citations (TC), h-index, average citations per paper (TC/TP), and the number of highly cited papers (≥100 citations and ≥10 citations), as well as their inclusion in the top 50 cited documents.

At the top of the list is Dinggang Shen from ShanghaiTech University, China, with 14 papers and 313 total citations, demonstrating strong productivity with an h-index of 10. However, authors such as Maryann E. Martone from the University of California, San Diego, and Hanchuang Peng from Janelia Research Campus, although having fewer papers (11 each), have a higher citation impact, with 462 and 355 total citations, respectively. Both have an h-index of 9, showing their influential work in the field. 

Giorgio A. Ascoli from George Mason University stands out with a high average citation rate (59.89 citations per paper) and 539 total citations from just 9 papers, placing him among the most impactful authors in Neuroinformatics. Similarly, Vince D. Calhoun from Georgia Institute of Technology has contributed 10 papers with an impressive citation rate of 34.10 per paper.

Several authors have papers with over 100 citations, indicating significant contributions to the field. Ascoli and Peter T. Fox have 2 papers with more than 100 citations, while Yu-Feng Zang from Hangzhou Normal University leads with 2,528 citations, an exceptionally high number that reflects the outsized influence of his work, despite contributing only 6 papers. Note that Peter T. Fox, from the University of Texas Health Science Center at San Antonio, is recognized by Clarivate Analytics as a Highly Cited Researcher in the field of Neuroscience and Behavior since 2015.

The authors come from a diverse range of institutions across the globe. The USA has a significant representation with institutions like University of California San Diego, Northwestern University, and Georgia Institute of Technology contributing leading researchers. China is also well-represented, with ShanghaiTech University, Nanjing Tech University, and Northwestern Polytechnical University making significant contributions, indicating the growing influence of Chinese research in Neuroinformatics.

**Table 7:** Top 35 leading authors in Neuroinformatics

*R*	Author name	University	Country	TP	TC	H	TC/TP	≥ 100	≥ 10	T50
1	Shen, D. G.	ShanghaiTech U	CHN	14	313	10	22.36	0	10	0
2	Martone, M. E.	U California San Diego	USA	11	462	9	42.00	1	8	2
3	Peng, H. C.	Janelia Research Campus	USA	11	355	9	32.27	0	8	1
4	Shepherd, G. M.	Northwestern U	USA	10	346	7	34.60	1	6	2
5	Calhoun, V. D.	Georgia Institute Tech	USA	10	341	6	34.10	1	3	0
6	Nowinski, W. L.	U Washington	USA	10	236	9	23.60	0	9	2
7	Ascoli, G. A.	George Mason U	USA	9	539	9	59.89	2	7	3
8	Grethe, J. S.	U California San Diego	USA	9	430	9	47.78	1	8	2
9	Wójcik, D. K.	Polish Academy Sciences	POL	9	240	8	26.67	0	8	0
10	Liu, T. M.	Nanjing Tech U	CHN	9	138	8	15.33	0	6	0
11	Turner, J. A.	The Ohio State U	USA	8	466	6	58.25	1	4	4
12	Toga, A. W.	U Southern California	USA	8	171	6	21.38	0	5	0
13	Guo, L.	Northwestern Polytech U	CHN	8	130	7	16.25	0	6	0
14	Zhang, H.	China Three Gorges U	CHN	7	461	6	65.86	1	3	1
15	Landman, B. A.	Vanderbilt U	USA	7	169	6	24.14	1	4	1
16	Zhang, D. Q.	Nanjing U Aeronautics Astron	CHN	7	125	5	17.86	0	5	0
17	Zhou, X. B.	UT Health Sci Center Houston	USA	7	98	4	14.00	0	4	0
18	Wong, S. T. C.	Houston Methodist Hospital	USA	7	90	4	12.86	0	3	0
19	Chen, R.	Johns Hopkins U	USA	7	44	4	6.29	0	2	0
20	Zang, Y. F.	Hangzhou Normal U	CHN	6	2,528	4	421.33	1	2	1
21	Marenco, L.	Yale U	USA	6	273	5	45.50	1	4	1
22	Miller, P. L.	Yale U	USA	6	213	4	35.50	1	3	1
23	Kennedy, D. N.	U Massachusetts Medical Sch	USA	6	184	4	30.67	1	2	1
24	Gong, H.	Huazhong U Science & Tech	CHN	6	87	6	14.50	0	5	0
25	Luo, Q. M.	Hainan U	CHN	6	87	6	14.50	0	5	0
26	Han, J. W.	Nanchang U	CHN	6	78	5	13.00	0	4	0
27	Defelipe, J.	CSIC	SPA	6	60	4	10.00	0	3	0
28	Fox, P. T.	UT Health Sci Center San Antonio	USA	5	461	3	92.20	1	2	1
29	Wang, Y.	Rensselaer Polytech Inst	USA	5	242	4	48.40	1	2	1
30	Li, Y.	Beihang U	CHN	5	86	4	17.20	0	3	0
31	Quan, T. W.	Guangxi U Chinese Medicine	CHN	5	73	5	14.60	0	4	0
32	Zeng, S. Q.	Huazhong U Science & Tech	CHN	5	73	5	14.60	0	4	0
33	Zhou, H.	Chengdu U Information Tech	CHN	5	73	5	14.60	0	4	0
34	Hu, X. T.	U Illinois Chicago	USA	5	67	4	13.40	0	3	0
35	Herskovits, E. H.	U Maryland Baltimore	USA	5	42	4	8.40	0	2	0

*C/P and H *= Cites per paper and h-index available in WoS

The most productive and influential institutions in the field of Neuroinformatics are outlined in detail, ranked by key metrics such as total publications (TP), total citations (TC), and h-index (H) in Table 8. Harvard University leads the rankings with 32 publications and 1,210 citations, achieving an h-index of 17 and a strong citations-per-paper (C/P) ratio of 37.81. Other top contributors include the University of California San Diego and the University of North Carolina Chapel Hill, which demonstrate significant impact through their research outputs and citation metrics. 

Institutions like Yale University and the University of Southern California also feature prominently, with high publication counts and influential papers in the field. University College London, as one of the top European institutions, stands out for its strong performance, with a high C/P ratio of 58.56 and contributions to cutting-edge research in Neuroinformatics.

The table reflects the global distribution of research excellence, featuring institutions from the United States, Europe, and China, and highlights the interdisciplinary collaboration that drives the field forward. Institutions like the National Institutes of Health (NIH) and the Polish Academy of Sciences also showcase significant research impact within Neuroinformatics, contributing to the development of this rapidly evolving field.

**Table 8:** The most productive and influential institutions in Neuroinformatics

*R*	Institution	Country	TP	TC	H	C/*P*	≥ 100	≥ 10	T50	QS	ARWU
1	Harvard U	USA	32	1,210	17	37.81	4	21	4	4	1
2	U California San Diego	USA	23	739	14	32.13	1	17	3	72	18
3	U NC Chapel Hill	USA	20	414	12	20.70	0	13	0	155	35
4	Yale U	USA	18	663	12	36.83	2	14	3	23	11
5	U Southern California	USA	18	408	11	22.67	1	12	1	125	62
6	U College London	UK	16	937	12	58.56	2	15	2	9	16
7	CNRS	FRA	16	254	9	15.88	0	9	1	-	-
8	Natl Inst Health (NIH)	USA	15	1,236	12	82.40	4	14	4	-	-
9	Massachusetts Gen Hosp	USA	14	834	11	59.57	3	11	4	-	-
10	U Pennsylvania	USA	13	758	8	58.31	2	7	3	11	14
11	U California Los Angeles	USA	13	306	9	23.54	0	8	0	42	15
12	INSERM	FRA	13	285	8	21.92	1	7	1	-	-
13	U Polytech Madrid	SPA	12	335	8	27.92	1	8	1	321	601-
14	Polish Academy Sciences	POL	12	259	9	21.58	0	9	0	-	-
15	UT Health Sci San Antonio	USA	11	697	6	63.36	1	6	3	-	-
16	Howard Hughes Medic Inst	USA	11	688	8	62.55	2	7	3	-	-
17	U Houston	USA	11	623	6	56.64	2	6	2	651-	201-
18	Radboud U Nijmegen	NET	11	370	7	33.64	1	7	1	272	101-
19	California Inst Technology	USA	10	550	7	55.00	2	6	2	10	8
20	George Mason U	USA	10	547	9	54.70	2	9	3	1001-	201-
21	Cornell U	USA	10	529	7	52.90	2	6	2	16	12
22	EPFL Lausanne	SWI	10	443	7	44.30	2	7	3	-	-
23	Allen Inst Brain Science	USA	10	259	8	25.90	0	7	0	-	-
24	Northwestern Polytech U	CHN	10	142	8	14.20	0	6	0	547	101-
25	10 institutions	-	10	-	-	-	-	-	-	-	-

*ARWU* Academic Ranking of World Universities, *QS* Quacquarelli & Symonds University Ranking

The most productive and influential countries in Neuroinformatics research are ranked based on various metrics, as outlined in Table 9. The United States leads with 314 papers, the highest number of citations (14,157), and an h-index of 51, underscoring its dominance in the field, with 23 papers cited over 100 times and 194 papers cited at least 10 times. The U.S. also boasts a high citation-per-paper ratio (C/P) of 45.09, highlighting the significant impact of its research. 

China ranks second with 86 papers and 3,911 citations, maintaining a high C/P of 45.48 and an h-index of 21. Although China's output is smaller than that of the U.S., it has steadily increased its influence, with two papers cited over 100 times and 46 papers cited more than 10 times. Germany and the United Kingdom follow closely, each contributing significantly with 52 and 58 papers, respectively. Germany shows a strong C/P of 36.63, while the UK has a C/P of 29.21, and both countries have multiple papers with over 100 citations. 

Switzerland, despite its smaller population, stands out for its high impact, with a remarkable C/P of 46.08 from 26 papers and an h-index of 12. It also has four papers cited over 100 times, highlighting its disproportionate influence in the field. Other European countries like the Netherlands, Italy, and Spain show strong contributions, while Canada remains a key player outside Europe and the U.S., with 30 papers and a C/P of 22.07.

In Asia, Japan and South Korea display notable contributions with high C/P ratios, while Singapore emerges as an influential country with a C/P of 20.08 despite its small size. Brazil and India, though producing fewer papers, show increasing influence, particularly Brazil, with a C/P of 44.64.

Countries such as Norway and Finland, although having lower outputs, display high C/Po ratios, indicating impactful research relative to their size. The data illustrates a diverse range of contributions globally, with North America and Europe leading in both productivity and influence in Neuroinformatics.

**Table 9:** Annual number of papers classified by countries

*R*	Country	TP	TC	H	C/*P*	≥ 100	≥ 10	T50	Population	*P*/Po	C/Po
1	USA	314	14,157	51	45.09	23	194	33	334,914,900	0.94	42.27
2	China	78	3,914	21	50.18	2	44	3	1,410,710,000	0.06	2.77
3	UK	58	1,694	20	29.21	3	36	5	68,350,000	0.85	24.78
4	Germany	52	1,905	20	36.63	5	33	5	84,482,270	0.62	22.55
5	Spain	41	670	11	16.34	2	16	2	48,373,340	0.85	13.85
6	Netherlands	34	831	14	24.44	3	20	3	17,879,490	1.90	46.48
7	Italy	33	974	13	29.52	4	15	4	58,761,150	0.56	16.58
8	Canada	30	662	13	22.07	3	18	3	40,097,760	0.75	16.51
9	France	30	562	12	18.73	1	16	2	68,170,230	0.44	8.24
10	Switzerland	26	1,198	12	46.08	4	15	5	8,849,850	2.94	135.37
11	Poland	25	461	13	18.44	0	16	0	36,685,850	0.68	12.57
12	Belgium	17	624	8	36.71	1	7	2	11,822,590	1.44	52.78
13	Australia	16	359	10	22.44	0	10	1	26,638,540	0.60	13.48
14	Japan	14	550	9	39.29	2	9	3	124,516,650	0.11	4.42
15	Singapore	13	261	9	20.08	0	9	0	5,917,650	2.20	44.11
16	Brazil	11	491	7	44.64	1	4	1	216,422,450	0.05	2.27
17	South Korea	10	306	8	30.60	1	7	1	60,414,500	0.17	5.07
18	India	10	180	6	18.00	0	5	0	1,428,627,660	0.01	0.13
19	Sweden	9	186	7	20.67	0	6	0	10,536,630	0.85	17.65
20	Finland	9	126	5	14.00	0	3	0	5,584,260	1.61	22.56
21	Norway	8	178	7	22.25	0	5	0	5,519,590	1.45	32.25
22	Taiwan	8	67	5	8.38	0	2	0	23,894,394	0.33	2.80
23	Denmark	6	83	4	13.83	0	2	0	5,946,950	1.01	13.96
24	Israel	6	68	3	11.33	0	3	0	9,756,700	0.61	6.97
25	Austria	6	25	3	4.17	0	0	0	9,132,380	0.66	2.74
26	Turkey	5	112	4	22.40	0	2	0	85,326,000	0.06	1.31
27	Iran	4	44	3	11.00	0	2	0	89,172,770	0.04	0.49
28	Portugal	4	23	2	5.75	0	1	0	10,525,350	0.38	2.19
29	Cuba	3	66	3	22.00	0	2	0	11,194,450	0.27	5.90
30	Greece	3	48	2	0.00	0	1	0	10,361,300	0.29	4.63
-	8 countries	2	-	-	-	-	-	-	-	-	-
-	10 countries	1	-	-	-	-	-	-	-	-	-

*P/Po* and *C/Po* = Papers and citations per million inhabitants

Table 10 provides a detailed breakdown of the annual number of papers published in Neuroinformatics by country, spanning from 2003 to 2023. The table highlights the total number of papers (TP) produced by each country, with further breakdowns of yearly contributions. 

The United States leads by a significant margin, with a total of 314 papers published over the period. The country consistently produced papers each year, peaking with 30 papers in 2022. This dominance reflects the United States's long-standing leadership in both neuroscience and computational sciences.

China ranks second with 78 papers, showing particularly strong output in recent years, with 11 papers published in 2022 and 10 in 2021, signifying the country's rapid rise in scientific contributions to Neuroinformatics. Germany and the United Kingdom follow with 52 and 58 papers, respectively, both displaying relatively steady output over the years.

Other notable contributors include Spain (41 papers), the Netherlands (34 papers), and Italy (33 papers), all of which have maintained consistent research activity in Neuroinformatics. Countries such as Poland (25 papers), Belgium (17 papers), and Australia (16 papers) also show meaningful contributions, while regions like South Korea, India, and Brazil have growing but smaller outputs.

Countries with lower total outputs, such as Israel, Austria, Turkey, and Iran, typically publish fewer than 10 papers, indicating room for growth in the field. Overall, Table 10 reflects both the dominance of historically strong research countries and the rising contributions of emerging nations in Neuroinformatics research.

**Table 10:** Annual number of papers classified by countries

*R*	Country	Total	23	22	21	20	19	18	17	16	15	14	13	12	11	10	09	08	07	06	05	04	03
1	United States	314	13	30	15	13	11	17	13	14	18	19	20	15	16	11	10	17	11	13	10	15	13
2	Peoples R China	78	3	11	10	8	5	11	6	4	2	3	7	1	1	0	1	3	0	0	0	1	1
3	United Kingdom	58	6	5	2	2	2	6	2	7	1	4	5	4	3	1	0	0	2	0	1	0	5
4	Germany	52	1	4	4	3	5	2	1	1	6	4	4	3	1	5	3	1	1	0	0	3	0
5	Spain	41	4	4	2	4	6	0	2	4	3	3	3	2	3	1	0	0	0	0	0	0	0
6	Netherlands	34	3	6	4	4	5	1	0	2	2	2	2	0	0	0	2	1	0	0	0	0	0
7	Italy	33	5	5	3	0	1	1	2	1	2	2	1	0	1	0	0	2	1	1	1	3	1
8	Canada	30	5	8	3	1	4	1	0	0	1	0	0	0	0	0	1	1	1	1	1	1	1
9	France	30	6	0	4	2	2	2	1	1	2	0	1	2	0	3	0	0	0	1	1	1	1
10	Switzerland	26	3	2	2	2	2	3	0	1	1	1	2	0	2	1	1	0	1	0	2	0	0
11	Poland	25	0	4	3	1	0	0	2	2	2	0	1	2	1	1	2	1	1	1	0	0	1
12	Belgium	17	1	1	4	0	0	3	0	1	1	0	2	0	0	1	0	0	2	0	0	0	1
13	Australia	16	0	2	0	2	2	1	0	3	0	1	0	1	0	1	0	0	1	1	0	1	0
14	Japan	14	1	2	2	0	1	0	0	0	0	0	0	0	2	1	1	1	1	0	2	0	0
15	Singapore	13	0	4	0	1	0	0	0	0	0	0	0	2	0	1	3	0	0	1	0	1	0
16	Brazil	11	0	1	0	1	2	1	0	0	1	0	1	1	0	1	0	0	1	0	0	0	1
17	South Korea	10	1	0	0	2	1	0	2	0	1	2	0	1	0	0	0	0	0	0	0	0	0
18	India	10	2	2	2	1	0	0	0	1	0	0	0	0	0	1	0	0	1	0	0	0	0
19	Sweden	9	0	1	1	0	1	1	1	1	0	1	0	1	0	1	0	0	0	0	0	0	0
20	Finland	9	3	0	0	3	1	0	0	1	0	0	0	0	0	0	0	0	0	1	0	0	0
21	Norway	8	0	1	2	0	0	0	1	0	1	0	0	0	1	0	0	0	1	0	0	1	0
22	Taiwan	8	0	0	1	1	0	2	1	0	0	1	1	0	1	0	0	0	0	0	0	0	0
23	Denmark	6	1	0	1	0	0	1	0	0	0	2	0	0	0	0	0	0	0	0	0	1	0
24	Israel	6	1	0	1	1	0	1	0	0	0	0	0	0	0	0	0	0	0	0	0	2	0
25	Austria	6	1	0	0	2	0	1	0	1	0	1	0	0	0	0	0	0	0	0	0	0	0
26	Turkey	5	0	3	0	1	0	0	0	0	0	1	0	0	0	0	0	0	0	0	0	0	0
27	Iran	4	2	0	0	1	0	0	0	0	0	0	0	0	1	0	0	0	0	0	0	0	0
28	Portugal	4	1	1	0	0	0	0	1	0	0	0	1	0	0	0	0	0	0	0	0	0	0
29	Cuba	3	0	0	0	0	1	0	0	0	0	0	0	0	0	1	0	0	0	0	0	1	0
30	Greece	3	2	0	0	0	1	0	0	0	0	0	0	0	0	0	0	0	0	0	0	0	0

23 − 03 = Annual number of articles between 2003 and 2023

The publication structure of Neuroinformatics research, classified by supranational regions, is detailed in Table 11. The table presents the total number of papers (TP), total citations (TC), h-index (H), citations per paper (C/P), and the number of highly cited papers (≥100 citations), as well as the number of papers cited at least 10 times (≥10). The data is normalized by population to show papers (P/Pop) and citations (C/Pop) per million inhabitants, providing a comparative view of research output across different regions.

North America leads with 332 papers, the highest number of total citations (14,718), and an h-index of 52, reflecting the region’s dominance in Neuroinformatics research. Europe follows with 270 papers and a total of 6,491 citations, with an h-index of 37. Both regions demonstrate strong research impact, with North America showing a higher number of highly cited papers (26 with ≥100 citations) and papers with more than 10 citations (205). Europe has 15 papers with over 100 citations and 146 papers with more than 10 citations, indicating significant research output but lower impact compared to North America.

Asia ranks third with 136 papers and 5,135 citations, driven primarily by contributions from Eastern Asia, which accounts for 102 papers. Asia's h-index of 29 and relatively high citation-per-paper ratio (37.76) demonstrate growing influence in the field, although the region trails in terms of highly cited papers, with only five papers exceeding 100 citations. Other regions, including the Middle East and the rest of Asia, show smaller contributions to the overall research output.

Latin America and Oceania contribute more modestly to the field, with 20 and 18 papers, respectively. While the total number of papers and citations is lower, both regions have demonstrated some highly cited work, with Latin America showing one paper with over 100 citations. Africa remains an emerging region in Neuroinformatics, with just three papers and minimal citations, reflecting the need for greater research development in this area. Overall, North America and Europe dominate the landscape, while Asia is steadily rising as a key contributor to global Neuroinformatics research.

**Table 11:** Publication structure classified by supranational regions

*R*	Region	TP	TC	H	C/*P*	≥ 100	≥ 10	T50	Population	*P*/Pop	C/Pop
1	North America	332	14,718	52	44.33	26	205	36	375,076,150	0.89	39.24
2	Europe	270	6,491	37	24.04	15	146	18	517,826,878	0.52	12.54
3	Asia	136	5,135	29	37.76	5	75	7	4,531,464,251	0.03	1.13
3.1	Eastern Asia	102	4,578	25	44.88	5	56	7	2,129,112,130	0.05	2.15
3.2	Rest of Asia	25	448	13	17.92	0	14	0	1,919,348,000	0.01	0.23
3.3	Middle East	16	236	8	14.75	0	8	0	483,004,121	0.03	0.49
4	Latin America	20	611	8	30.55	1	8	1	659,310,564	0.03	0.93
5	Oceania	18	379	10	21.06	0	11	1	31,861,640	0.56	11.90
6	Africa	3	48	2	16.00	0	2	0	1,523,367,712	0.00	0.03

*P/Po and C/Po* = Papers and cites per million inhabitants

## Mapping Neuroinformatics with VOS viewer software

In order to reach a more detailed level of understanding of the literature review of the Neuroinformatics bibliometric material, we develop a graphical mapping of this data using the VOSviewer software (Van Eck and Waltman 2010). For more information regarding the VOSviewer and how to use it, see Van Eck and Waltman (2023), and the webpage of the software: https://www.vosviewer.com.

### Co-citation analysis in Neuroinformatics

First, we analyse co-citation of cited journals in Neuroinformatics, which we find when two published articles receive a citation in a third paper published in Neuroinformatics (Small 1973). Figure 4 presents a cocitation network of journals in Neuroinformatics, constructed using VOSviewer with a minimum citation threshold of 20 and 100 links between the journals. This visualization highlights the interconnectedness of various scientific publications cited by and frequently co-cited alongside Neuroinformatics.

One of the key observations from the network is the central role of Neuroimage, which appears as the most prominent node. This indicates its dominant position in neuroimaging and computational neuroscience research. Neuroimage exhibits strong co-citation links with a variety of other journals, reflecting its broad influence across medical imaging, brain mapping, and related neuroscience disciplines.

In contrast, Neuroinformatics holds a significant, though somewhat smaller, position in the network. It is connected to important journals like Frontiers in Neuroinformatics, PLOS One, and Scientific Reports, underscoring its relevance in computational biology, bioinformatics, and data analysis. The proximity of these journals suggests a close relationship between Neuroinformatics and fields that leverage computational tools for neuroscience research.

The network reveals distinct clusters of related fields. The green cluster, centred around Neuroimage, focuses on journals in neuroimaging and medical imaging, such as Magnetic Resonance Imaging and Human Brain Mapping. Meanwhile, the red cluster highlights core neuroscience journals like Journal of Neuroscience, Nature Neuroscience, and Neuron, indicating the fundamental role of these publications in the development of neuroscience. A blue cluster surrounding Neuroinformatics includes journals related to computational neuroscience and bioinformatics, showcasing the journal’s contributions to bridging neuroscience with computational methods.

Prominent general science and neuroscience journals, such as Nature, Science, and Proceedings of the National Academy of Sciences (PNAS), also have strong co-citation ties with Neuroinformatics. This emphasizes the journal’s integration into high-impact, cutting-edge research across multiple disciplines.

**Fig. 5:**
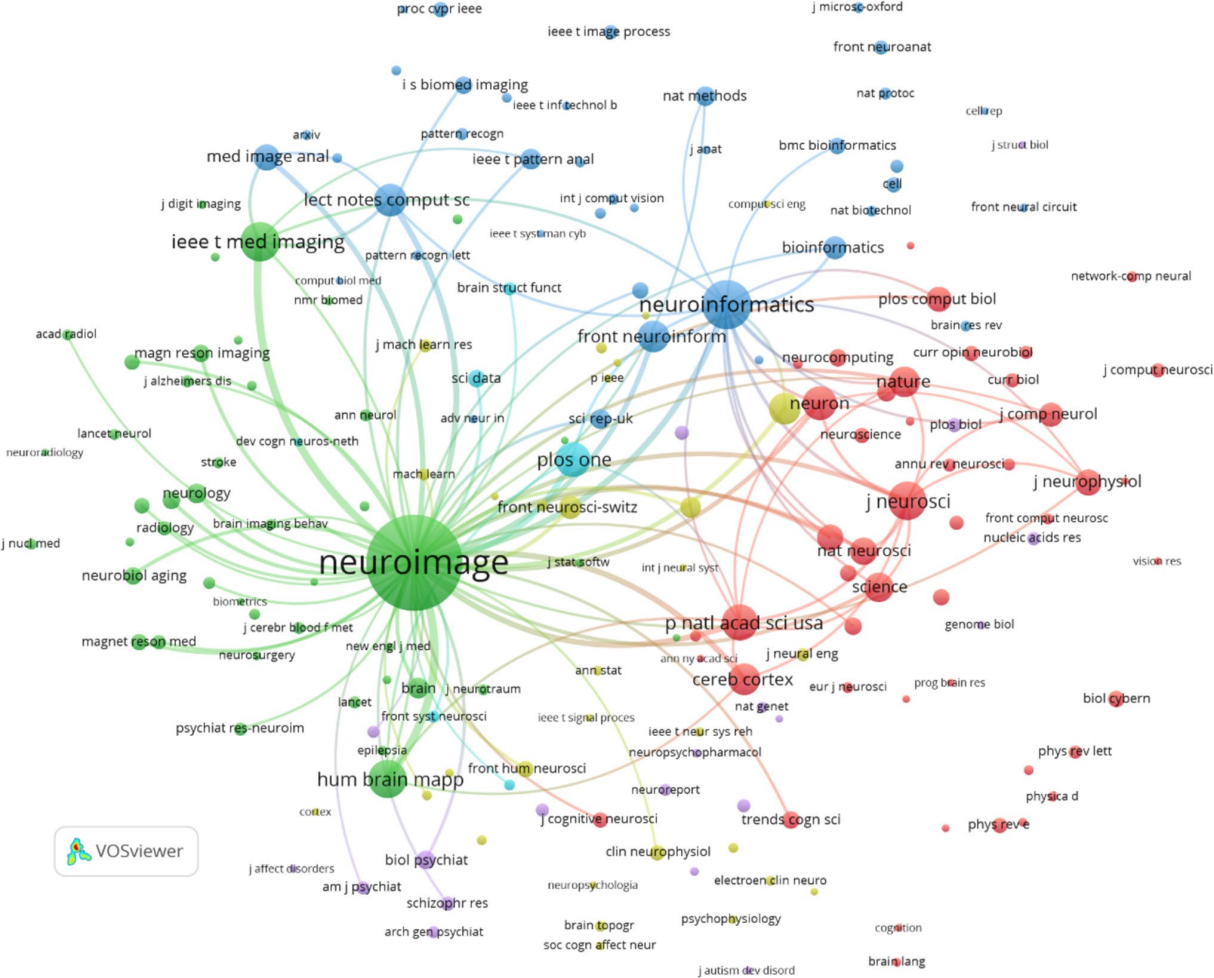
Co-citation of journals in Neuroinformatics: minimum citation threshold of 20 and 100 links

Overall, Figure 5 demonstrates the interdisciplinary reach of Neuroinformatics. Its close ties with journals in computer science, engineering, and bioinformatics—like IEEE Transactions on Medical Imaging and PLOS Computational Biology—reflect the journal’s pivotal role in advancing computational approaches in brain science. The cocitation patterns underscore how Neuroinformatics acts as a bridge between neuroscience, computational tools, and data-driven methodologies, contributing significantly to the field’s growth and development.

**Fig. 6:**
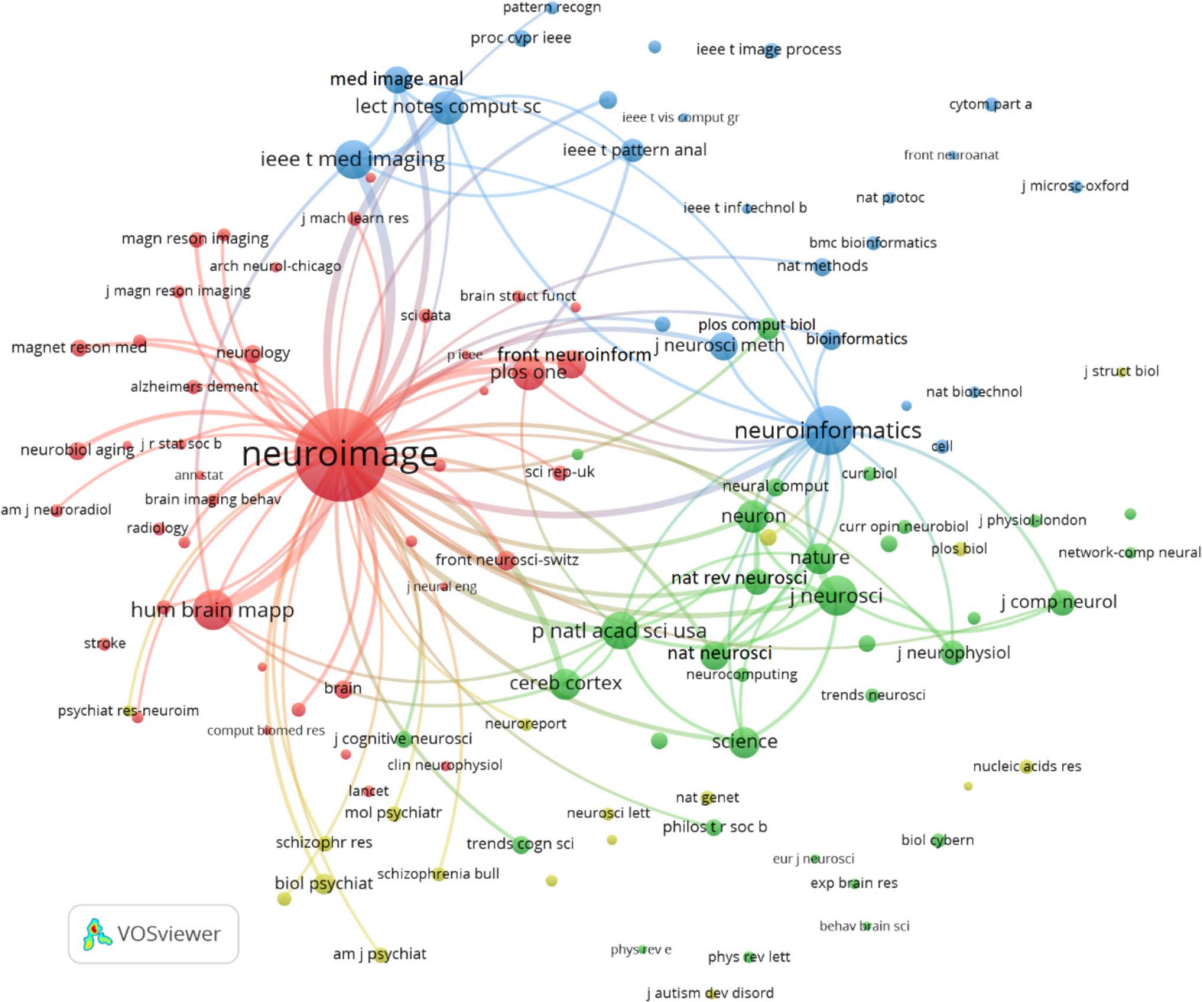
Co-citation of journals in Neuroinformatics: North America (minimum citation threshold of 20 and 100 links)

Figure 6 illustrates the co-citation network of journals related to Neuroinformatics articles from North America, constructed using VOSviewer with a minimum citation threshold of 20 and 100 links. This network visualizes the relationships between journals frequently co-cited alongside Neuroinformatics, highlighting the field's interdisciplinary nature and collaborative ties.

One of the most prominent features of the network is the dominance of Neuroimage, which is the largest and most central node. Neuroimage plays a pivotal role in the neuroimaging and neuroscience communities, evidenced by its strong co-citation links with numerous journals. These links include key publications like Human Brain Mapping, Magnetic Resonance in Medicine, and Radiology, all of which emphasize the central importance of imaging research in neuroscience and its frequent collaboration with other medical imaging fields.

Neuroinformatics also holds a significant position within the network, though it is smaller in comparison to Neuroimage. It is tightly linked to journals such as PLOS One, Frontiers in Neuroinformatics, Bioinformatics, and Scientific Reports, demonstrating its role in the computational and bioinformatics domains. These connections suggest that Neuroinformatics plays a crucial role in the development of computational tools, data analysis, and bioinformatics in neuroscience. 

The network is composed of distinct clusters that reveal different areas of focus. The red cluster around Neuroimage is heavily centred on neuroimaging, featuring journals like Human Brain Mapping and Magnetic Resonance Imaging, while the green cluster represents foundational neuroscience journals such as Journal of Neuroscience, Nature Neuroscience, and Proceedings of the National Academy of Sciences (PNAS). The blue cluster focuses on computational and bioinformatics journals, with Neuroinformatics linking to Bioinformatics, IEEE Transactions on Medical Imaging, and other computational science journals, highlighting its interdisciplinary nature.

Additionally, journals like Nature, Science, and PNAS show strong connections within the network, suggesting that Neuroinformatics and related journals are frequently co-cited with high-impact, general science publications. This underscores the journal’s broad interdisciplinary reach, bridging both fundamental neuroscience research and applied computational methods.

The network also reflects a strong connection between computational and clinical fields. Journals like PLOS Computational Biology and IEEE Transactions on Medical Imaging are closely linked to clinical publications such as Radiology and Neurology, illustrating the dynamic relationship between computational advancements and their practical applications in clinical neuroscience and medical imaging.

In conclusion, Figure 6 highlights the broad and interdisciplinary influence of Neuroinformatics within the North American research community. Neuroimage emerges as a central player in neuroimaging, while Neuroinformatics bridges the computational and bioinformatics fields with neuroscience. The co-citation patterns reveal the journal’s significant role in advancing both computational tools and clinical applications, emphasizing its importance in a wide array of scientific fields.

**Fig. 7:**
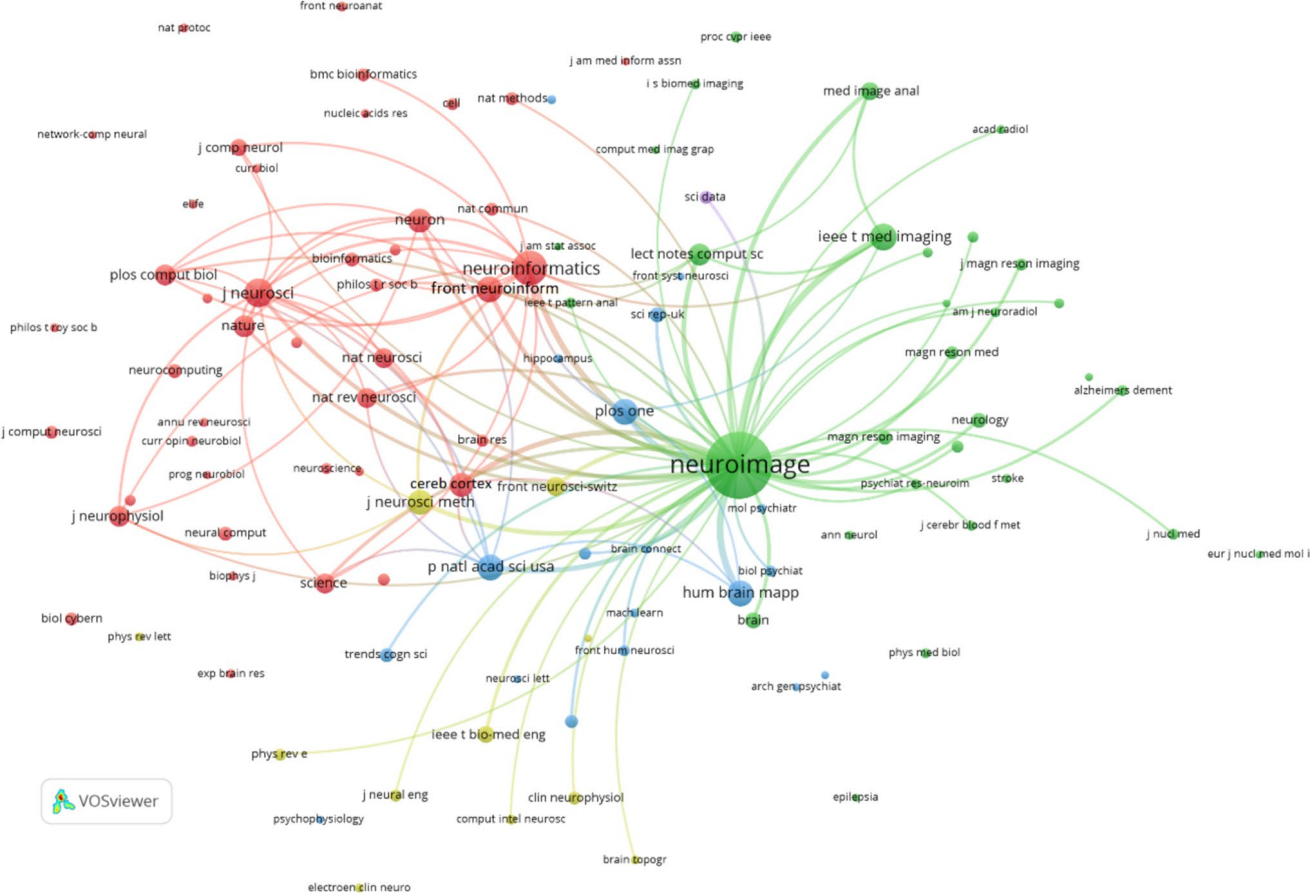
Co-citation of journals in Neuroinformatics: Europe (minimum citation threshold of 20 and 100 links)

Figure 7 provides a visualization of the co-citation network among journals involved in Neuroinformatics research across Europe, generated using VOSviewer with a minimum citation threshold of 20 and 100 links. This network illustrates the relationships between journals frequently co-cited alongside Neuroinformatics, offering insight into the interdisciplinary nature of the field within European research.

One of the most prominent observations is the centrality of Neuroimage, which once again stands out as the most dominant node in the network. Neuroimage plays a crucial role in neuroimaging and neuroscience research, demonstrating strong co-citation links with journals such as Human Brain Mapping, Magnetic Resonance Imaging, and IEEE Transactions on Medical Imaging. These connections emphasize the vital role of imaging and radiology in European neuroscience, with Neuroimage acting as a bridge between medical, radiological, and computational journals.

Neuroinformatics occupies a smaller yet significant role in the network, particularly in the computational and bioinformatics domains. Journals such as Frontiers in Neuroinformatics, PLOS One, and Bioinformatics are closely linked to Neuroinformatics, indicating its importance in computational neuroscience and interdisciplinary collaboration within Europe. These connections underscore the growing reliance on data analysis, bioinformatics, and computational tools in neuroscience research.

The network reveals several distinct clusters of journals. The green cluster centres around Neuroimage and focuses primarily on neuroimaging, featuring journals like Magnetic Resonance Imaging and IEEE Transactions on Medical Imaging, which highlight the integration of advanced imaging techniques into neuroscience research. The red cluster includes foundational neuroscience journals such as Journal of Neuroscience, Nature Neuroscience, Neuron, and PLOS Computational Biology, which are essential for advancing basic neuroscience knowledge. The blue cluster comprises computational and informaticsfocused journals like Bioinformatics and Lecture Notes in Computer Science, reflecting the significance of computational methodologies in European Neuroinformatics research.

The interdisciplinary nature of the field is further emphasized by the presence of high-impact general science journals such as Nature, Science, and Proceedings of the National Academy of Sciences (PNAS). These journals frequently appear alongside core neuroscience and computational publications, indicating that Neuroinformatics research bridges the gap between fundamental neuroscience and applied computational innovations.

Additionally, the network reveals strong connections between clinical journals, such as Neurology and Stroke, and computational publications like PLOS Computational Biology. This suggests a dynamic relationship between computational advancements and their applications in clinical neuroscience within European research, demonstrating how computational tools are directly impacting clinical practices.

**Fig. 8:**
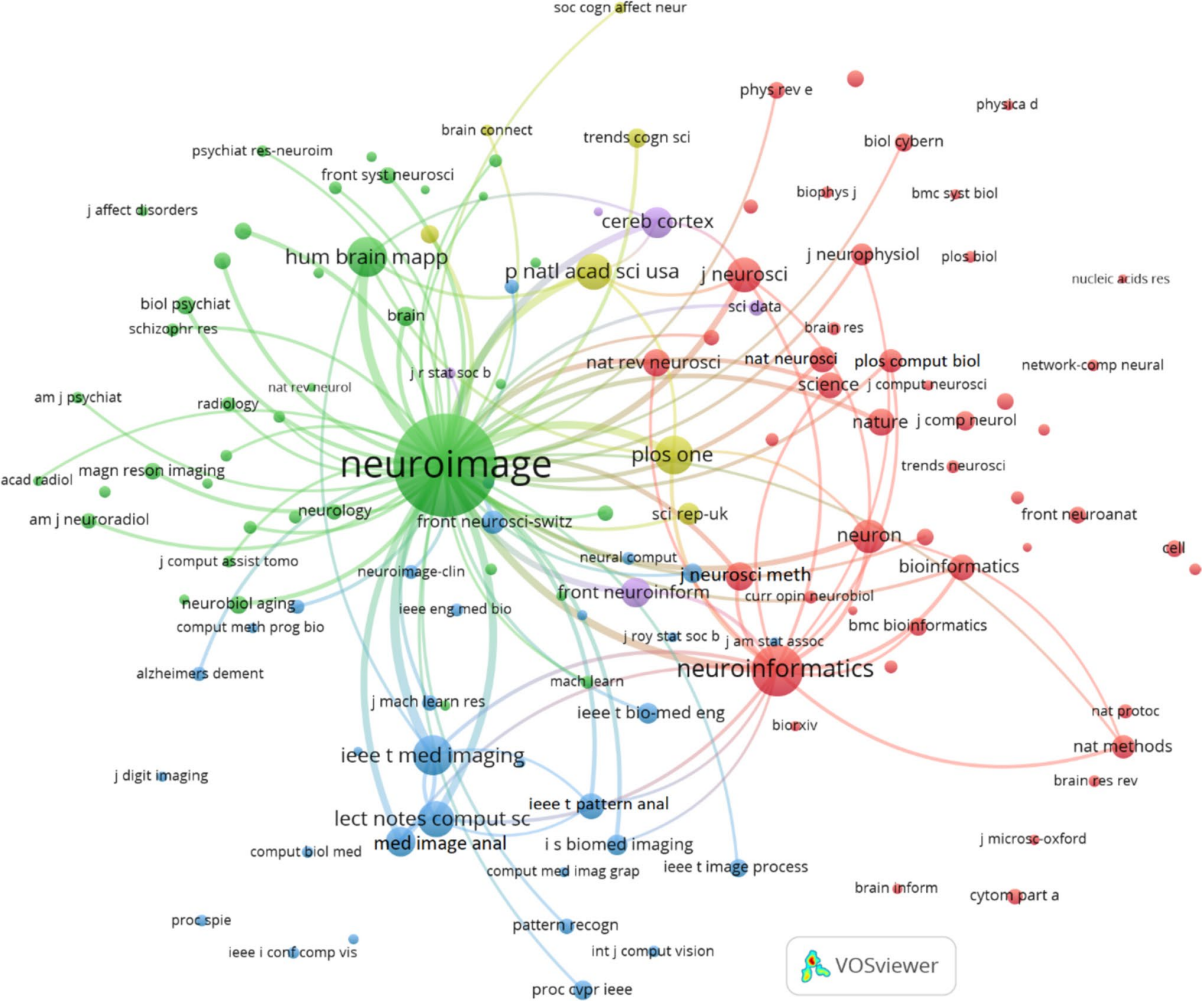
Co-citation of journals in Neuroinformatics: Rest of the World (minimum citation threshold of 20 and 100 links)

Figure 8 displays the co-citation network of journals related to Neuroinformatics research from the "Rest of the World" (excluding Europe and North America), created using VOSviewer with a minimum citation threshold of 20 and 100 links. This network visualizes how journals frequently co-cited with Neuroinformatics connect, offering a glimpse into the interdisciplinary collaborations and research contributions from regions outside the traditional Western academic hubs.

Neuroimage emerges as the most prominent and central node, reflecting its influential role in global neuroimaging research. Its extensive cocitation links with journals like Human Brain Mapping, Magnetic Resonance Imaging, and IEEE Transactions on Medical Imaging emphasize its strong association with both neuroimaging and related medical technologies. Serving as a major hub, Neuroimage connects various domains within neuroimaging, medical imaging, and radiology.

Neuroinformatics holds a key position within the network, particularly through its connections to journals that specialize in computational neuroscience and bioinformatics. Journals such as Frontiers in Neuroinformatics, Bioinformatics, PLOS Computational Biology, and PLOS One are closely linked, illustrating Neuroinformatics’ integral role in advancing computational tools, data analysis methods, and bioinformatics approaches in neuroscience research on a global scale.

Distinct clusters appear in the network, each highlighting different areas of focus. The green cluster revolves around Neuroimage and includes journals like Human Brain Mapping and Neurology, primarily centred on neuroimaging and radiology. Meanwhile, the red cluster focuses on foundational neuroscience and computational journals, including Journal of Neuroscience, Neuron, Nature Neuroscience, and PLOS Computational Biology, showcasing the interplay between computational methods and core neuroscience research. The blue cluster highlights the connection between computational technologies and neuroscience, featuring journals like IEEE Transactions on Medical Imaging, Medical Image Analysis, and Lecture Notes in Computer Science.

The interdisciplinary reach of Neuroinformatics in this region is further demonstrated by the strong co-citation links between high-impact, general science journals such as Nature, Science, and Proceedings of the National Academy of Sciences (PNAS) and core neuroscience and computational publications. This suggests that computational neuroscience extends beyond traditional neuroscience into broader scientific and technological fields.

Moreover, there are significant co-citation relationships between clinical journals like Neurology and Radiology and computational journals such as Bioinformatics and PLOS Computational Biology. This highlights a close connection between computational advances and their practical applications in clinical neuroscience and medical practices across regions outside of Europe and North America.

Table 12 provides a detailed global and temporal analysis of the cocitation trends within Neuroinformatics, ranking the most frequently cited journals across four time periods: the overall global analysis, 2019–2023, 2014–2018, and 2003–2013. This breakdown offers insights into the evolving influence of key journals within the field of computational neuroscience, neuroimaging, and related disciplines.

Across the entire period, Neuroimage consistently ranks first, with a total of 3,287 citations, underscoring its enduring importance in neuroimaging research. This reflects Neuroimage's central role in the development of imaging technologies and methods used in neuroscience. Neuroinformatics holds a strong second position globally, with 880 citations, affirming its pivotal role in computational neuroscience and the development of data-driven approaches. Other significant journals include IEEE Transactions on Medical Imaging (546 citations) and Human Brain Mapping (538 citations), highlighting the continuous relevance of imaging and brain mapping technologies across the global research landscape. Foundational journals like Journal of Neuroscience (537 citations) and Proceedings of the National Academy of Sciences (PNAS) (472 citations) demonstrate their broad influence in neuroscience research.

In the most recent period (2019–2023), Neuroimage remains the dominant journal, with 1,467 citations, continuing its trend of high influence in neuroimaging. Neuroinformatics retains its importance, accumulating 254 citations, while Human Brain Mapping (237 citations) and PLOS One (237 citations) also show robust citation counts, illustrating the growing trend towards interdisciplinary and open-access research. A notable shift is the rise of Frontiers in Neuroinformatics, which garnered 216 citations during this period, reflecting an increasing interest in interdisciplinary approaches to computational neuroscience. Emerging journals like Neuron (192 citations) and Scientific Reports (124 citations) also show significant citation activity, indicating their expanding influence in cutting-edge neuroscience research.

During 2014–2018, Neuroimage continued to dominate with 931 citations, demonstrating its sustained leadership in neuroimaging studies. Neuroinformatics remained a key journal with 271 citations, reflecting its continued role in advancing computational methodologies. IEEE Transactions on Medical Imaging (187 citations) and Lecture Notes in Computer Science (158 citations) highlight the growing integration of computational techniques into neuroscience. This period also saw the increased influence of PLOS One (150 citations) and the emergence of Med Image Analysis (82 citations) as critical journals for researchers focusing on image processing in neuroscience.

In the earliest period of analysis (2003–2013), Neuroimage had 744 citations, affirming its foundational role in shaping the neuroimaging field. Neuroinformatics played a critical role in establishing computational approaches in neuroscience with 339 citations during this time. Traditional neuroscience journals like Journal of Neuroscience (218 citations) and PNAS (185 citations) were also highly influential, contributing to the theoretical and methodological advancements in early Neuroinformatics research. Key neuroscience journals like Cerebral Cortex (139 citations) and Neuron (112 citations) were instrumental in driving interdisciplinary research between computational tools and core neuroscience topics. 

Across all periods, Neuroimage and Neuroinformatics maintain their status as the leading journals, reflecting the essential role of neuroimaging and computational approaches in the broader field of neuroscience. Recent periods (2019–2023) have shown a growing emphasis on interdisciplinary collaboration, as indicated by the rising influence of open-access journals like PLOS One and the increased citations for Frontiers in Neuroinformatics. In earlier periods (2003–2013), foundational neuroscience journals like Journal of Neuroscience and PNAS played a key role in shaping early Neuroinformatics research, highlighting the enduring impact of these traditional journals in the field.

**Table 12:** Co-citation of journals in Neuroinformatics: global and temporal analysis

	Global		2019–2023		2014–2018		2003–2013	
R	Journal	Cit	Journal	Cit	Journal	Cit	Journal	Cit
1	Neuroimage	3,287	Neuroimage	1,467	Neuroimage	931	Neuroimage	744
2	Neuroinformatics	880	Neuroinformatics	254	Neuroinformatics	271	Neuroinformatics	339
3	IEEE T Med Imaging	546	Hum Brain Mapp	237	IEEE T Med Imaging	187	J Neurosci	218
4	Hum Brain Mapp	538	PLOS One	237	PLOS One	150	P Natl Acad Sci USA	185
5	J Neurosci	537	Front Neuroinform	216	Lect Notes Comput Sc	127	Hum Brain Mapp	162
6	P Natl Acad Sci USA	472	J Neurosci	199	Hum Brain Mapp	124	Science	159
7	PLOS One	436	Neuron	192	J Neurosci	110	IEEE T Med Imaging	156
8	Neuron	405	IEEE T Med Imaging	187	Front Neuroinform	100	J Comp Neurol	144
9	Lect Notes Comput Sc	392	P Natl Acad Sci USA	186	Neuron	91	Cereb Cortex	139
10	Cereb Cortex	371	J Neurosci Meth	167	P Natl Acad Sci USA	87	Neuron	112
11	Front Neuroinform	371	Nature	164	J Neurosci Meth	86	J Neurophysiol	111
12	J Neurosci Meth	365	Lect Notes Comput Sc	158	Med Image Anal	82	Nature	109
13	Nature	338	Front Neurosci	147	Cereb Cortex	79	J Neurosci Meth	108
14	Science	313	Cereb Cortex	143	Bioinformatics	72	Lect Notes Comput Sc	99
15	Nat Neurosci	281	Nat Neurosci	135	IEEE T Pattern Anal	68	Nat Rev Neurosci	97
16	Med Image Anal	255	Plos Comput Biol	131	Nature	60	Nat Neurosci	87
17	Nat Rev Neurosci	254	Sci Rep-UK	124	PLOS Comput Biol	60	Philos T R Soc B	70
18	J Neurophysiol	249	Med Image Anal	110	Nat Rev Neurosci	55	Biol Cybern	67
19	PLOS Comput Biol	233	Sci Data	102	J Neurophysiol	54	Neural Comput	64
20	J Comp Neurol	221	Nat Methods	98	Science	53	Neurocomputing	62
21	Front Neurosci-Switz	199	Nat Rev Neurosci	96	Neurology	50	Med Image Anal	58
22	Bioinformatics	193	Science	93	Magn Reson Med	49	Brain Res	56
23	IEEE T Pattern Anal	163	Nat Commun	86	Nat Neurosci	48	IEEE T Pattern Anal	56
24	IEEE T Bio-Med Eng	160	J Neurophysiol	78	BMC Bioinformatics	46	IEEE T Bio-Med Eng	55
25	Brain	155	Front Hum Neurosci	72	Brain	46	Phys Rev E	55
26	Sci Rep-UK	148	IEEE T Bio-Med Eng	72	Proc Cvpr IEEE	43	Biol Psychiat	51
27	Nat Methods	145	Bioinformatics	68	Magn Reson Imaging	40	J Am Med Inform Assn	51
28	Neurology	138	Brain	64	I S Biomed Imaging	38	Phys Rev Lett	45
29	Neural Comput	127	Neuroimage-Clin	59	Trends Cogn Sci	38	J Cognitive Neurosci	43
30	Biol Psychiat	122	Cell	57	Neurobiol Aging	36	Magnet Reson Med	42

*R* Rank, *Cit* Citations

**Fig. 9:**
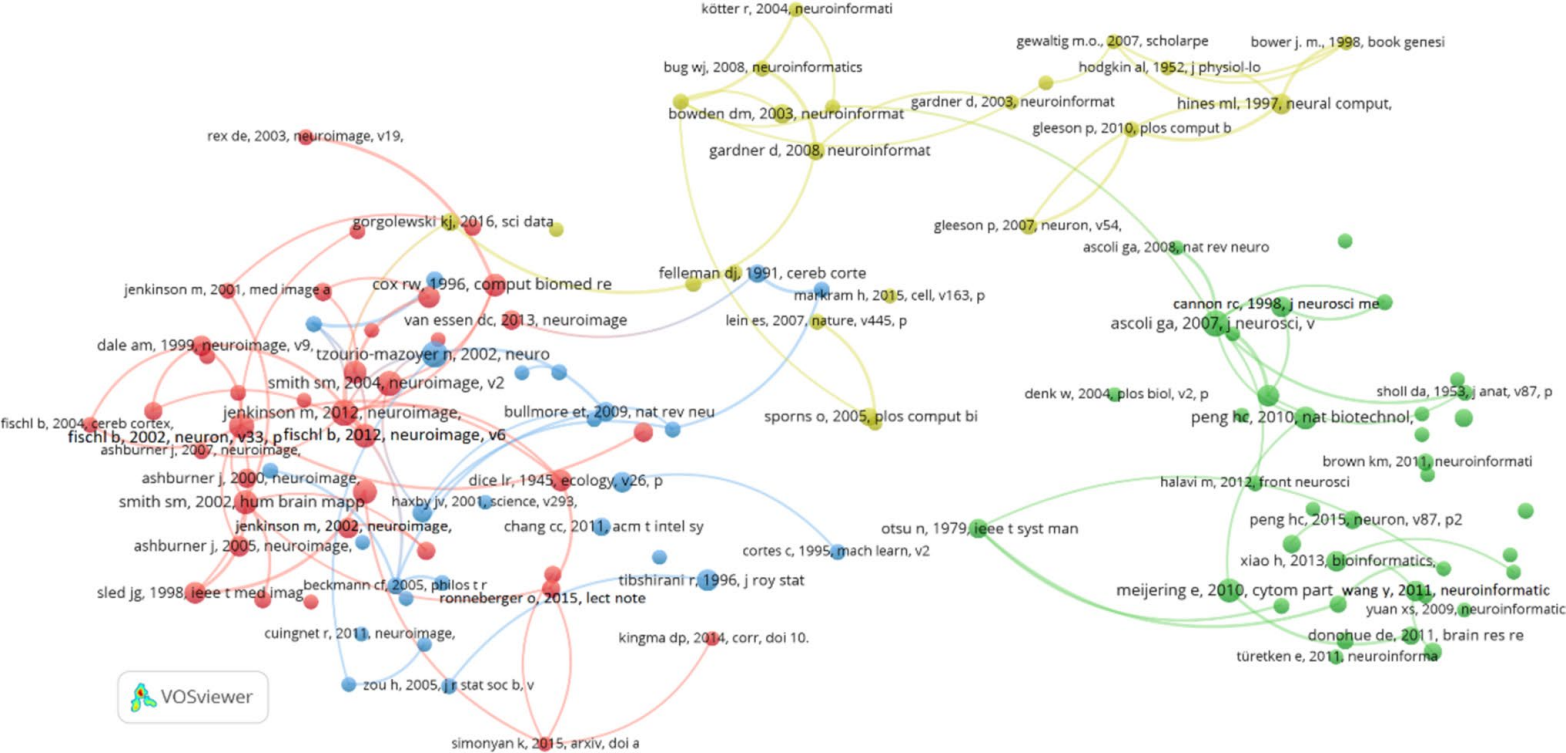
Co-citation of documents in Neuroinformatics: minimum citation threshold of 10 and 100 links

The co-citation network of documents in Neuroinformatics shown in Figure 9 was created using VOSviewer with a minimum citation threshold of 10 and 100 links between documents. This network highlights how frequently key papers are co-cited in Neuroinformatics, offering insights into the most influential works and their thematic connections within the field.

One prominent observation is the dominance of papers focused on methodological advancements, particularly in neuroimaging and computational neuroscience. Papers authored by Smith et al. (2002), Fischl et al. (2002) and Fischl (2012), which discuss widely-used neuroimaging tools such as FSL and FreeSurfer, are central in this network. These methodological papers are foundational to the development of imaging processing in neuroscience and form strong cocitation connections with other important works related to neuroimaging data analysis.

The network reveals several distinct clusters representing different research themes. The red cluster is heavily centred around neuroimaging methodologies, featuring key papers by Smith, Fischl, and Ashburner. These works are highly influential in the field, particularly for their contributions to the development of imaging software and analysis platforms like FreeSurfer. The blue cluster includes documents related to statistical and computational methods, such as Otsu (1979) on image processing techniques and Tibshirani (1996) on the Lasso regression method. These computational techniques have been widely applied in neuroimaging research and data analysis, contributing to their frequent co-citation with imaging-focused papers.

Another important area within the network is represented by the green cluster, which focuses on brain connectivity, neuron tracing, and bioinformatics tools. Influential papers in this group include works by Peng et al. (2010, 2015) on neuron tracing and brain connectivity. These contributions highlight the growing integration of computational tools into research on neural networks and connectivity, bridging the gap between traditional neurobiology and advanced data-driven analysis techniques.

The network underscores the interconnectedness of neuroimaging research, with strong links between papers on imaging tools and software. For example, documents by Jenkinson et al. (2012) and Cox (1996) are frequently co-cited, reflecting their central roles in developing neuroimaging processing pipelines. These co-citation patterns indicate that imaging software, such as FSL and AFNI, is integral to neuroscience research, facilitating large-scale data analysis and processing.

In addition, several key papers within the green and yellow clusters focus on bioinformatics and Neuroinformatics platforms. Papers by Gardner et al. (2008) and Bowden and Dubach (2003) explore Neuroinformatics data platforms, highlighting the importance of data-sharing systems in neuroscience research. These connections demonstrate the interdisciplinary nature of the field, where computational tools and bioinformatics resources are essential for advancing neuroscience discoveries.

**Fig. 10:**
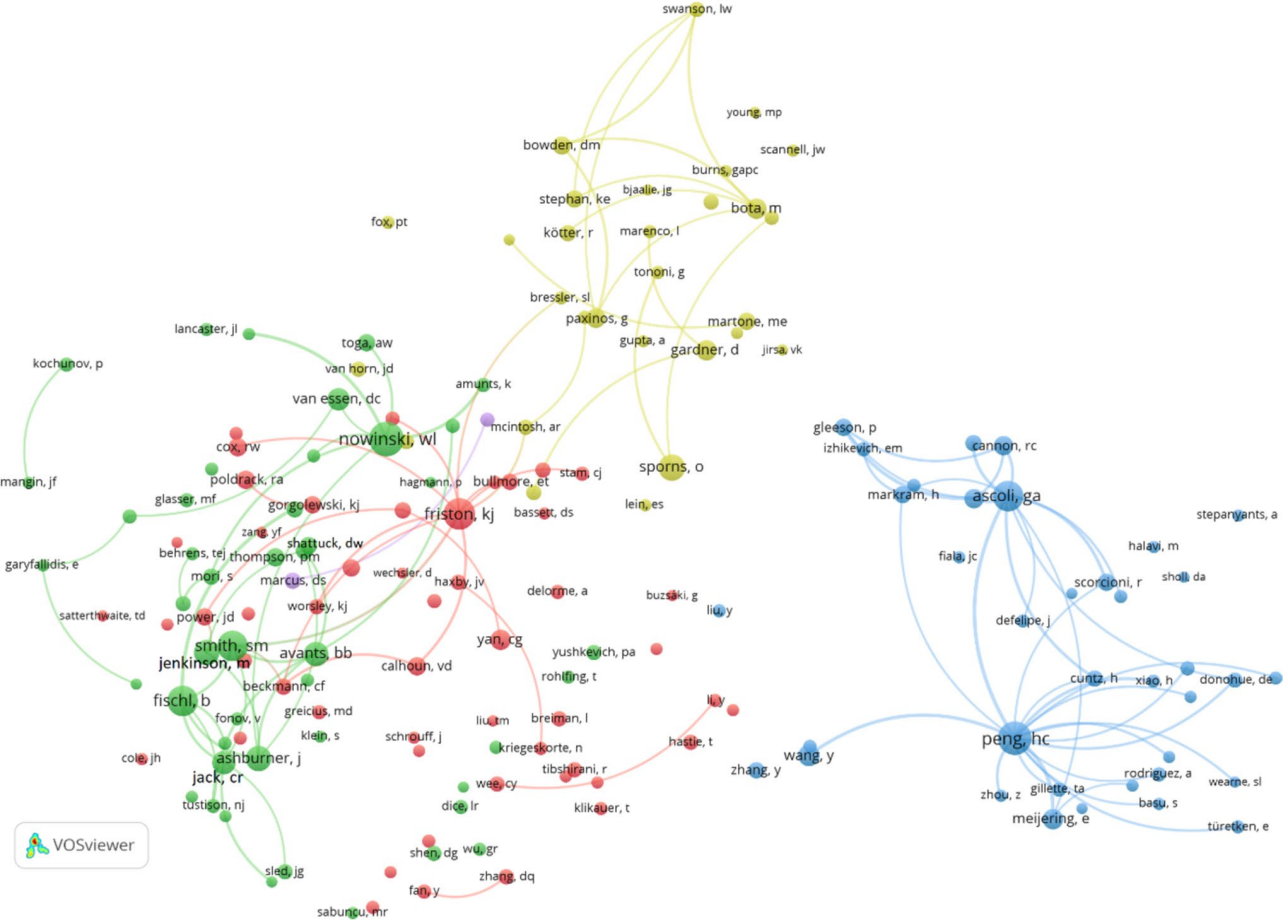
Co-citation of authors in Neuroinformatics: minimum citation threshold of 15 and 100 links

The co-citation network of key authors in Neuroinformatics is depicted in Figure 10, generated using VOSviewer with a minimum citation threshold of 15 and 100 links. This network shows how often certain authors are cited together, offering insights into major contributors and thematic areas within the field.

Several clusters emerge, each representing different areas of research. The green cluster is dominated by authors like Smith, Fischl, and Jenkinson, who are well-known for their work in neuroimaging and the development of tools such as FSL and FreeSurfer. These authors are frequently co-cited due to their contributions to the core methodologies used in neuroimaging analysis.

The yellow cluster features authors like Sporns and Bullmore, who are prominent in the study of brain connectivity. Their research focuses on mapping brain networks and understanding how different regions interact, making their work foundational in the field of network neuroscience.

In the blue cluster, authors such as Peng and Ascoli are key figures. Their work is centred around neuron tracing and the development of computational tools for analysing brain structures. These contributions have advanced the field of computational neuroanatomy, and they are frequently co-cited in studies focused on neural data processing.

The red cluster includes Friston, who has made significant contributions to statistical modeling in neuroimaging, particularly in the analysis of fMRI data. His work is widely cited, reflecting its importance in the development of statistical approaches to understanding brain function.

### Bibliographic coupling in Neuroinformatics

**Fig. 11:**
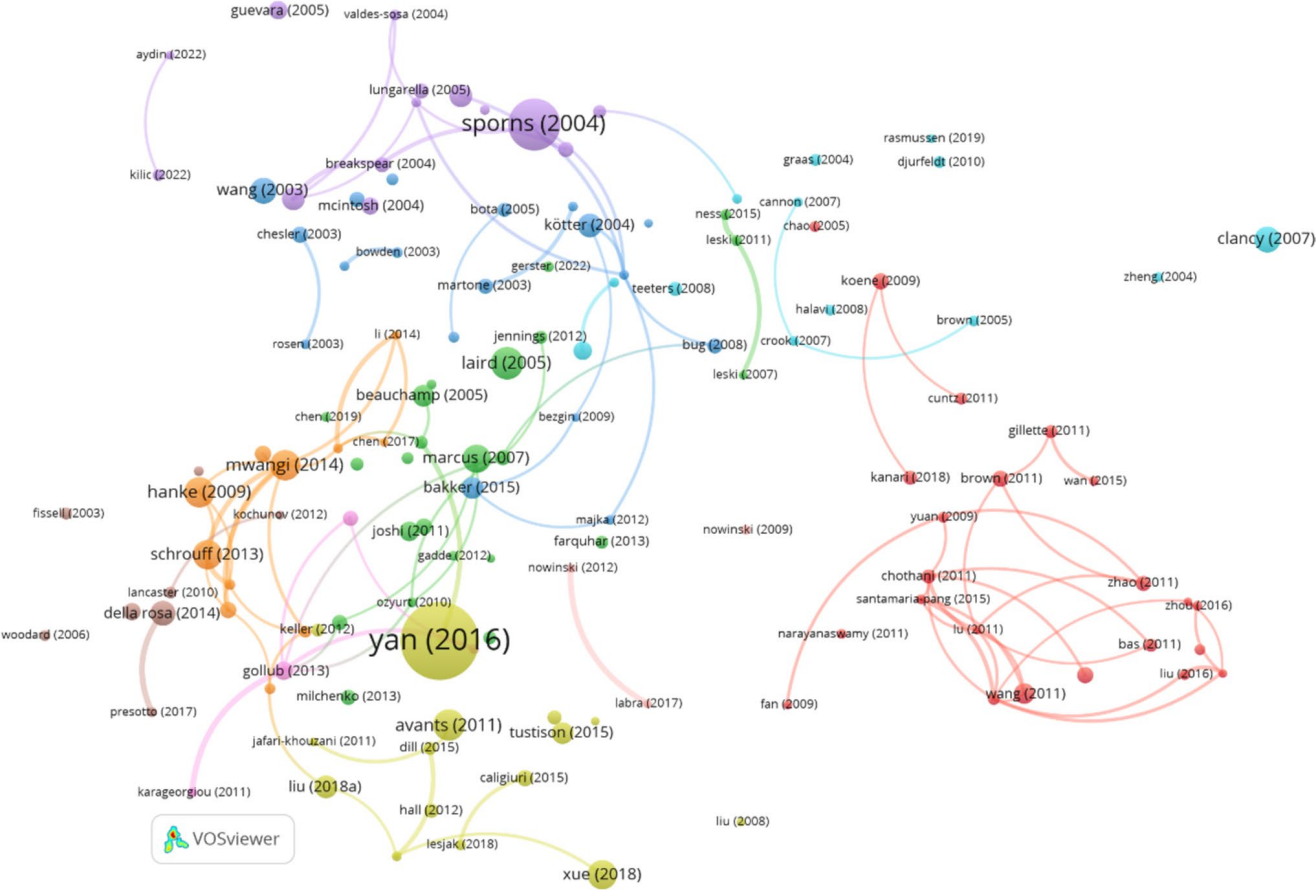
Bibliographic coupling of documents published in Neuroinformatics: minimum threshold of 20 citations and 100 links

The bibliographic coupling network of documents in Neuroinformatics is depicted in Figure 11, using a minimum threshold of 20 citations and 100 links. This network reveals how research papers are connected through shared references, providing a detailed view of thematic connections and the influence of various publications within the field. 

A standout feature in the network is the dominant position of Yan (2016), which is highly connected to other papers across multiple research areas. This indicates its significant influence, particularly in advancing research on brain imaging and data processing. The central role of Yan (2016) in the network highlights its broad application and its foundational contributions to the ongoing development of Neuroinformatics.

The network also identifies several distinct research clusters. In the green cluster, key works by Laird (2005) focus on neuroimaging techniques such as brain mapping and imaging analysis. These papers contribute significantly to the refinement of imaging methodologies and are frequently linked through shared references. Meanwhile, the orange cluster, featuring documents like Mwangi (2014) and Hanke (2009), centres on advanced neuroimaging techniques, especially the use of multivariate pattern analysis in fMRI data, further enriching the field of neuroimaging.

In the blue and purple clusters, we see a focus on brain connectivity and data sharing, with influential contributions from Sporns (2004) and Kötter (2004). Their work on mapping the brain's structural and functional networks has had a profound impact on the field, reflected in the strong coupling between their research and other studies in Neuroinformatics platforms.

The red cluster emphasizes computational neuroscience, with papers like Wang (2011) and Chothani (2011) driving advancements in machine learning and data analysis for brain data. These works highlight the growing trend of integrating computational methods to analyse largescale neural datasets.

**Fig. 12:**
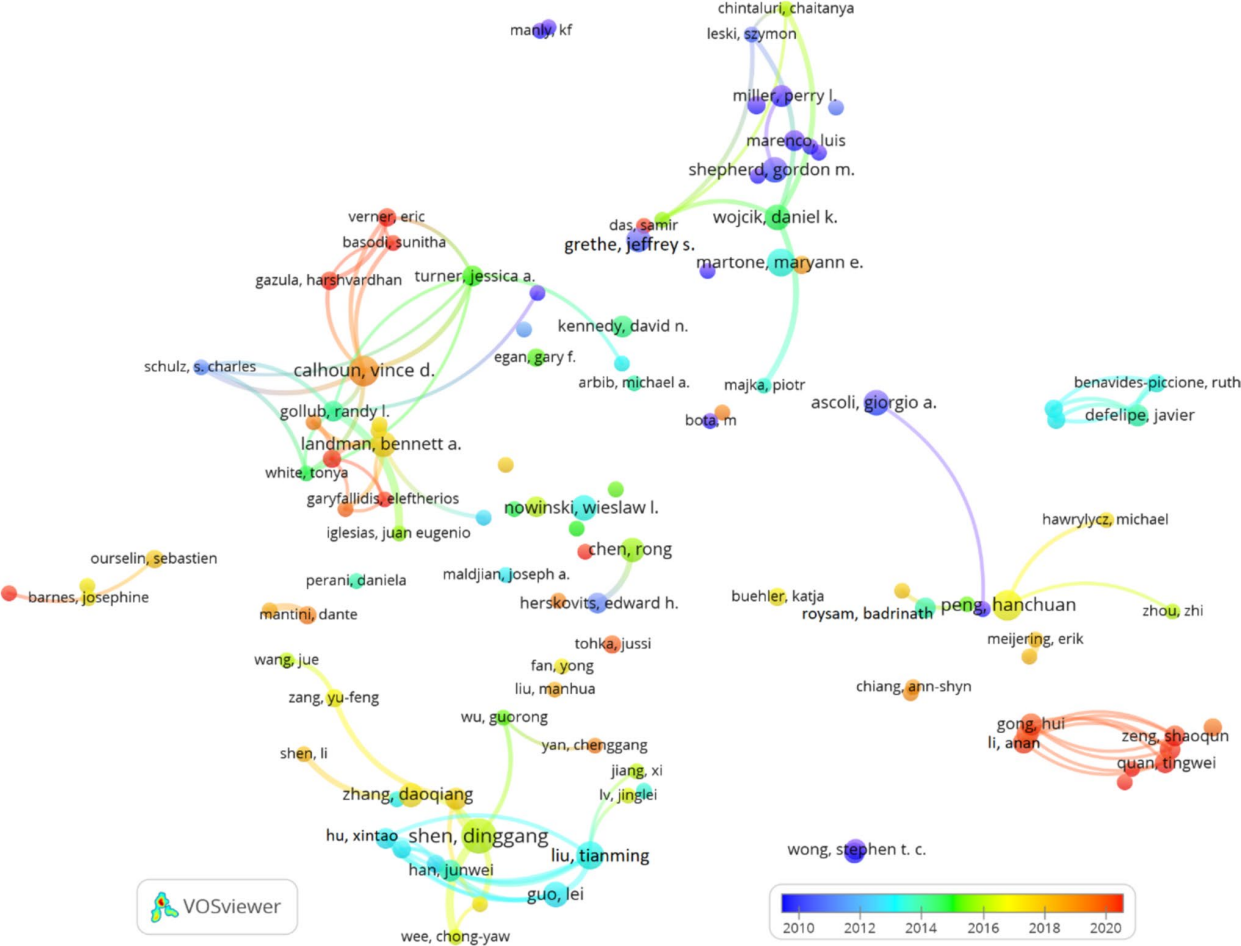
Bibliographic coupling of authors publishing in Neuroinformatics: minimum threshold of 3 documents and 100 links

Figure 12 provides a bibliographic coupling network of authors publishing in Neuroinformatics, based on a minimum threshold of 3 documents and 100 links. This visualization showcases how authors are connected through shared references in their published works, offering valuable insights into collaboration patterns and research focus areas within the field.

A number of central figures stand out in the network, including Vince D. Calhoun, Bennett A. Landman, and Dinggang Shen. These authors are prominent in the areas of neuroimaging and computational neuroscience, with extensive connections to other researchers in the field. Their strong presence in the network reflects their critical role in advancing imaging techniques and brain mapping tools, and their wide-ranging collaborations indicate their influence in shaping research directions in these areas.

The network also reveals distinct clusters of collaboration among various groups of researchers. One notable cluster includes Calhoun and Landman, who are linked with other influential figures like Randy Gollub and Tonya White. This suggests a concentrated focus on neuroimaging and brain connectivity research within this group. Another important cluster centres around Giorgio A. Ascoli, who collaborates closely with Hanchuan Peng and other researchers, reflecting their work on neuron tracing and computational neuroanatomy.

The timeline-based colour gradient in the network highlights emerging trends in the field. Authors such as Dinggang Shen and Daoqiang Zhang appear in more recent publications, indicating their involvement in cutting-edge advancements in neuroimaging and computational neuroscience. Similarly, Hanchuan Peng has been active in recent years, contributing significantly to the development of neuron tracing tools and bioinformatics platforms, reflecting the evolving landscape of Neuroinformatics research.

Furthermore, interdisciplinary collaborations are evident in the network, showcasing how researchers from different fields come together to address complex challenges in neuroscience. For instance, authors like Jeffrey S. Grethe, Maryann E. Martone, and Gordon M. Shepherd are part of a cluster that bridges Neuroinformatics with data-sharing platforms. Their work underscores the importance of interdisciplinary efforts in advancing computational methods and fostering innovation in neuroscience.

**Fig. 13:**
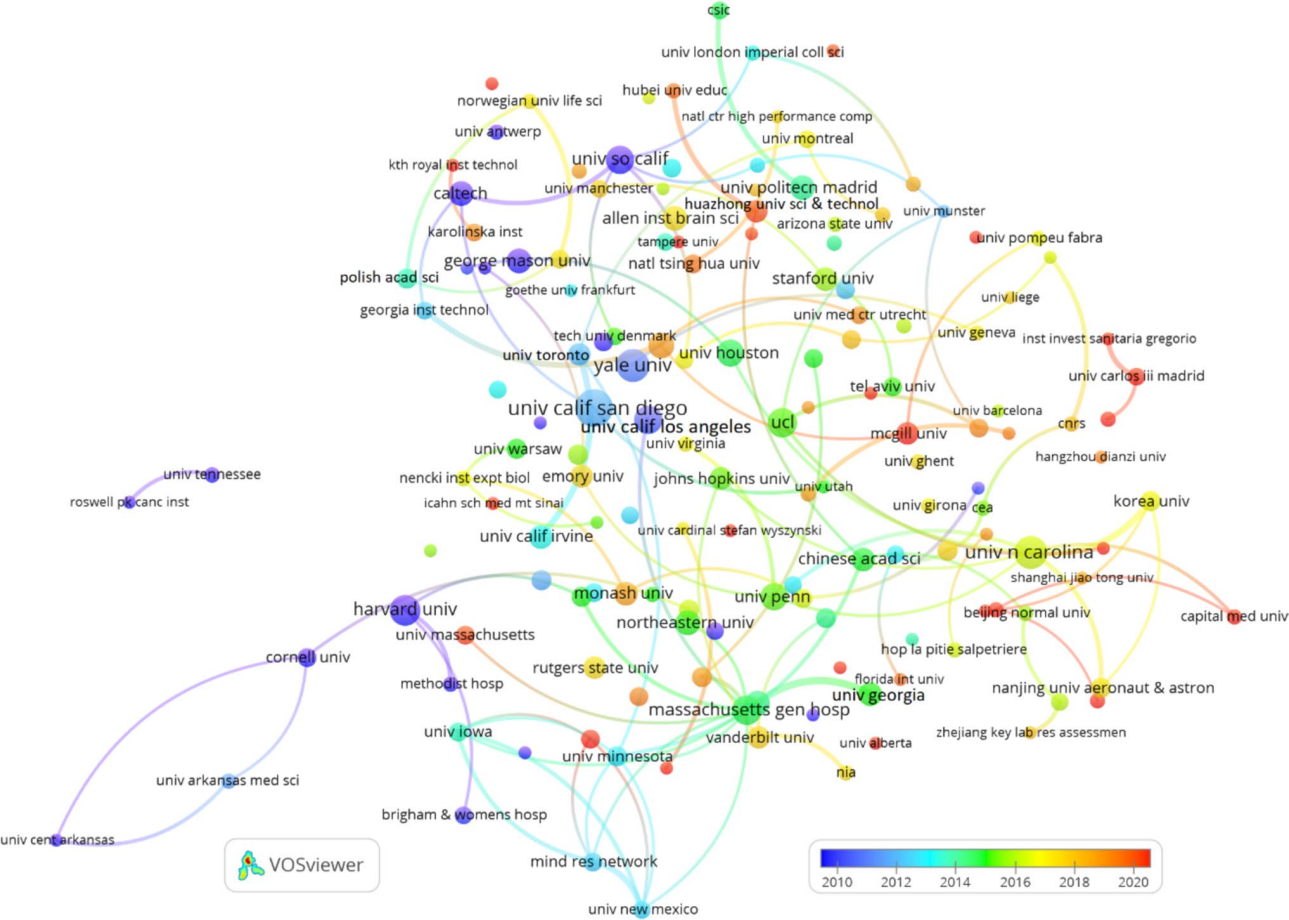
Bibliographic coupling of institutions publishing in Neuroinformatics: minimum publication threshold of 3 documents and 100 links

Figure 13 presents the bibliographic coupling network of institutions publishing in Neuroinformatics, with a minimum publication threshold of 3 documents and 100 links. The network illustrates how various institutions are connected based on shared references in their research publications, shedding light on collaboration patterns and the influence of different institutions within the field.

Several key institutions emerge as central figures in the network, notably University of California San Diego, Massachusetts General Hospital, Chinese Academy of Sciences, and Harvard University. These institutions are heavily involved in Neuroinformatics research, and their extensive connections to other organizations underscore their significant role in shaping the field through collaborative research and shared thematic interests.

The network also reveals distinct clusters of collaboration between institutions. For example, University of California San Diego, Yale University, and Stanford University form a strong collaborative group, which appears to focus on advanced neuroimaging and computational neuroscience. Another notable cluster includes Harvard University, Cornell University, and University of Arkansas Medical Sciences, suggesting shared research efforts, likely centred on computational techniques and medical applications of Neuroinformatics. 

International collaborations are another key feature of the network, with European institutions like University College London (UCL), Université Paris-Saclay, and University of Barcelona closely linked to U.S. institutions such as Johns Hopkins University and University of North Carolina. This global interconnectedness highlights the international scope of Neuroinformatics research. Additionally, Asian institutions like Chinese Academy of Sciences and Korea University are well integrated into this collaborative network, indicating the growing involvement of these regions in cutting-edge computational neuroscience.

The colour gradient in the network reflects the timeline of research activity, with more recent collaborations and research contributions coming from institutions like McGill University, University Carlos III Madrid, and Shanghai Jiao Tong University. These institutions are at the forefront of recent advancements in Neuroinformatics, particularly in areas like data processing, brain mapping, and neuroimaging techniques.

**Fig. 14:**
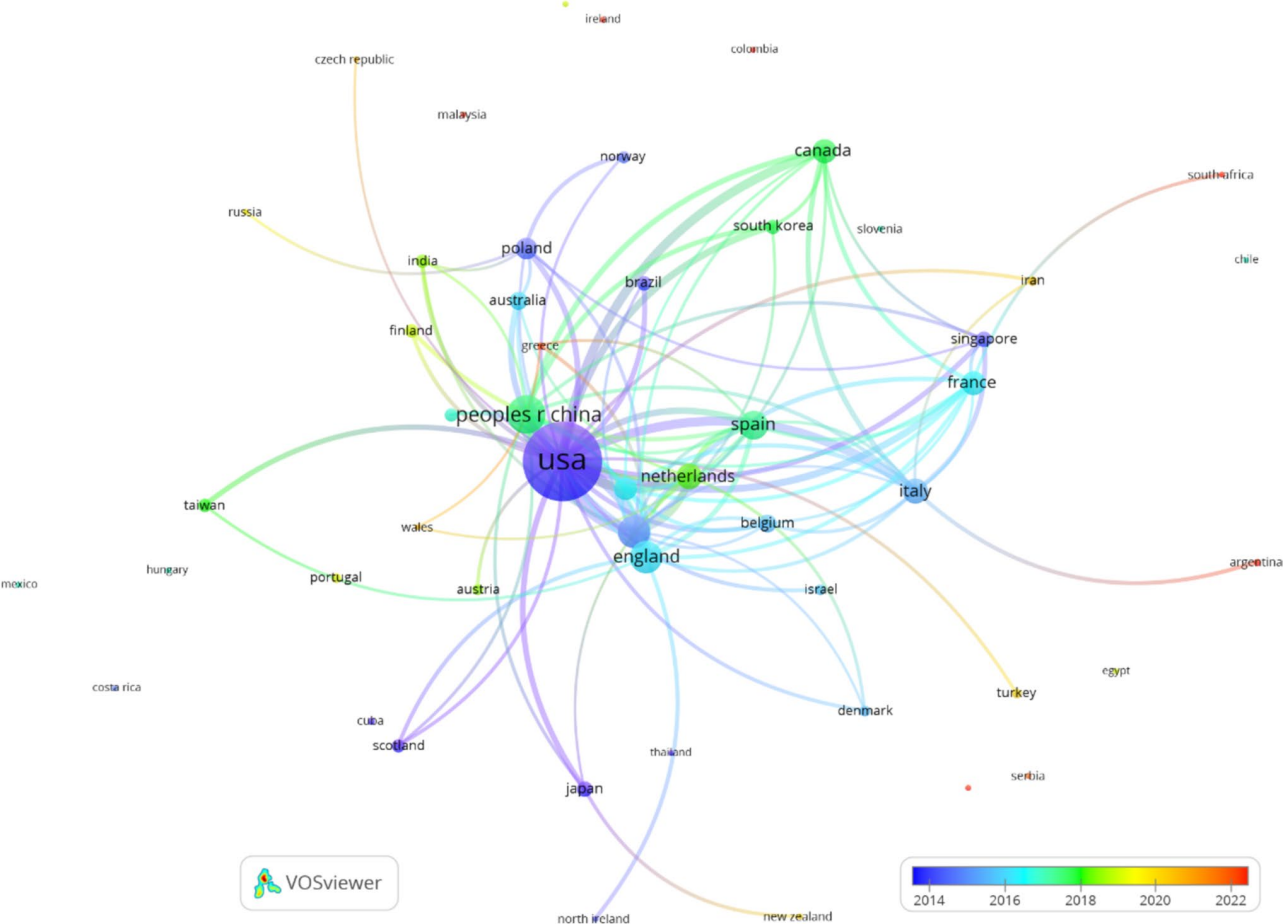
Bibliographic coupling of countries publishing in Neuroinformatics: minimum publication threshold of 1 document and 100 links

The bibliographic coupling network of countries involved in Neuroinformatics research is shown in Figure 14, using a minimum threshold of 1 document and 100 links. This visualization highlights how countries are connected through shared references in their publications, offering insights into the global research landscape and collaborations within the field of Neuroinformatics.

At the centre of the network, the USA and People’s Republic of China stand out as major hubs. These two countries have the most connections to other nations, underscoring their dominant roles in shaping global research efforts in Neuroinformatics. The USA, in particular, is highly interconnected, forming collaborative links with nearly every country in the network, highlighting its leadership in fostering international research collaborations.

European countries like England, Netherlands, Spain, France, and Italy also feature prominently in the network, reflecting their active participation in Neuroinformatics research. These nations show strong cross-border collaborations with each other as well as with the USA and China, making Europe a key player in global research efforts. In Asia, South Korea, Japan, and Singapore emerge as important contributors, demonstrating the growing role of East Asia in computational neuroscience and Neuroinformatics. 

The network also reveals emerging collaborations from countries like India, Australia, and Brazil. These nations show increased research activity in recent years, as indicated by the colour gradient in the visualization, reflecting their growing involvement in global Neuroinformatics research. Their connections to well-established research hubs suggest a rising influence in the field.

Though North America, Europe, and East Asia dominate the network,other regions are also beginning to contribute more significantly. Countries such as Mexico, South Africa, Argentina, and Turkey are part of the broader research landscape, indicating the expanding geographic reach of Neuroinformatics research. While they may not be as central as the leading countries, their inclusion in the network demonstrates the growing international diversity in the field.

### Keyword and topical analysis

**Fig. 15:**
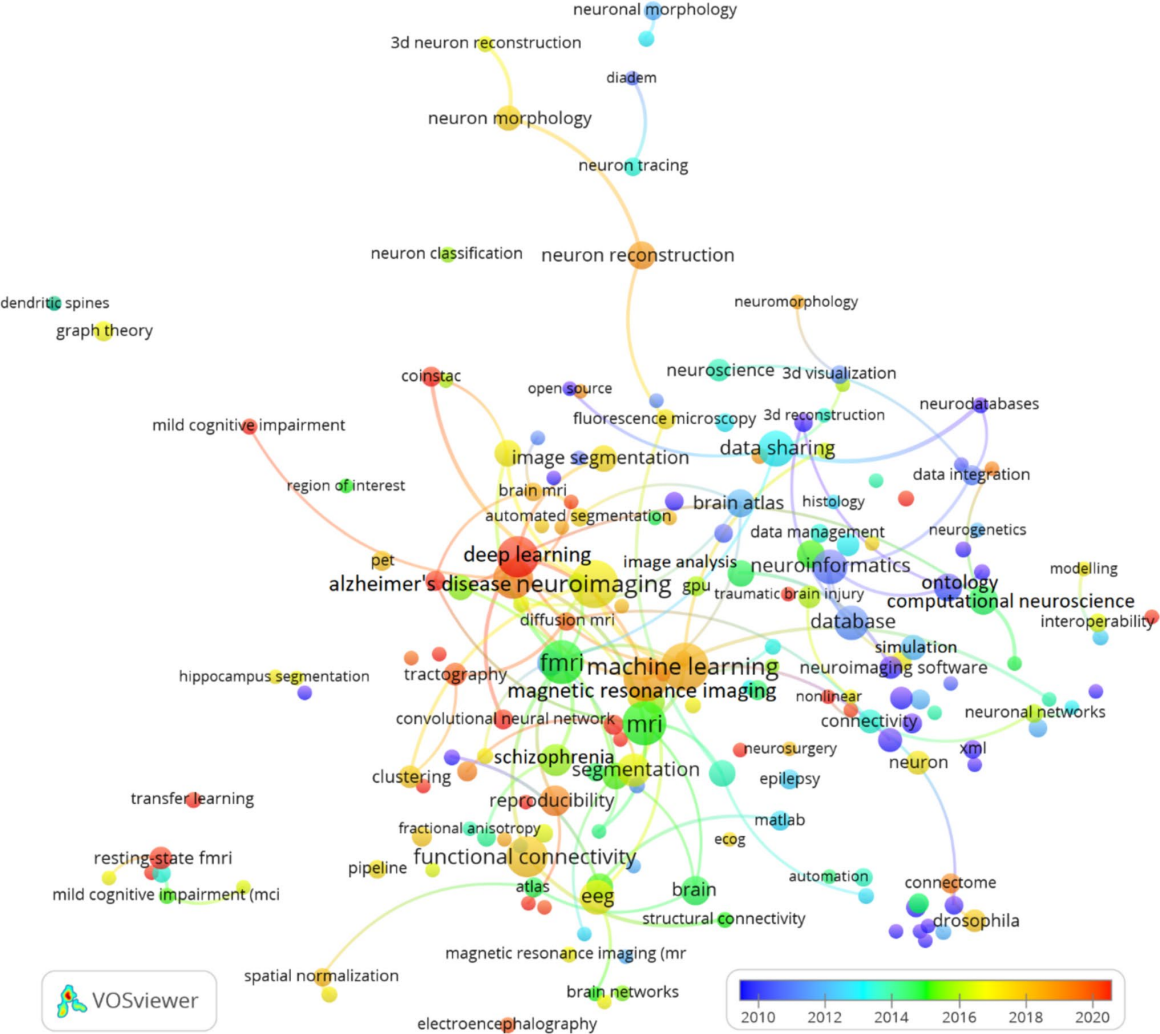
Co-occurrence of author keywords in Neuroinformatics: minimum occurrence threshold of 3 and 100 links

The co-occurrence network of author keywords in Neuroinformatics is depicted in Figure 15, using a minimum occurrence threshold of 3 and 100 links. This visualization illustrates the most frequently used keywords in research articles and their interconnections, providing valuable insights into the main research themes and emerging trends within the field.

At the core of the network is neuroimaging, which is closely associated with other significant topics such as fMRI, machine learning, and magnetic resonance imaging (MRI). This central position reflects the prominence of neuroimaging, particularly MRI and fMRI (Filippi, 2025), in Neuroinformatics research, with machine learning playing a pivotal role in processing and analysing neuroimaging data.

A notable trend in the network is the increasing integration of deep learning and image segmentation techniques, particularly in the context of Alzheimer’s disease and brain MRI. This highlights the growing use of advanced machine learning methods to analyse complex brain data, with a strong focus on applying these technologies to study neurodegenerative disorders.

The terms functional connectivity and brain networks also appear prominently, emphasizing research aimed at understanding the interactions between different regions of the brain. This focus is further supported by related terms such as tractography and diffusion MRI, indicating a strong interest in mapping brain connectivity using advanced imaging techniques. Additionally, keywords such as data sharing, informatics, and database point to the increasing importance of open data practices and the use of databases in Neuroinformatics. These terms underscore the collaborative nature of the field, particularly in large-scale brain mapping and neuroimaging studies.

Another important research area involves neuron morphology, 3D neuron reconstruction, and neuron tracing, which are associated with efforts to map the physical structure of neurons. These terms highlight the critical role of Neuroinformatics tools in anatomical neuroscience and neuron-level analysis. Other research areas, such as schizophrenia, epilepsy, and mild cognitive impairment, are also represented in the network, reflecting ongoing studies into neurological and psychiatric disorders. These terms are linked to keywords like functional connectivity and machine learning, indicating a computational approach to understanding these conditions.

**Fig. 16:**
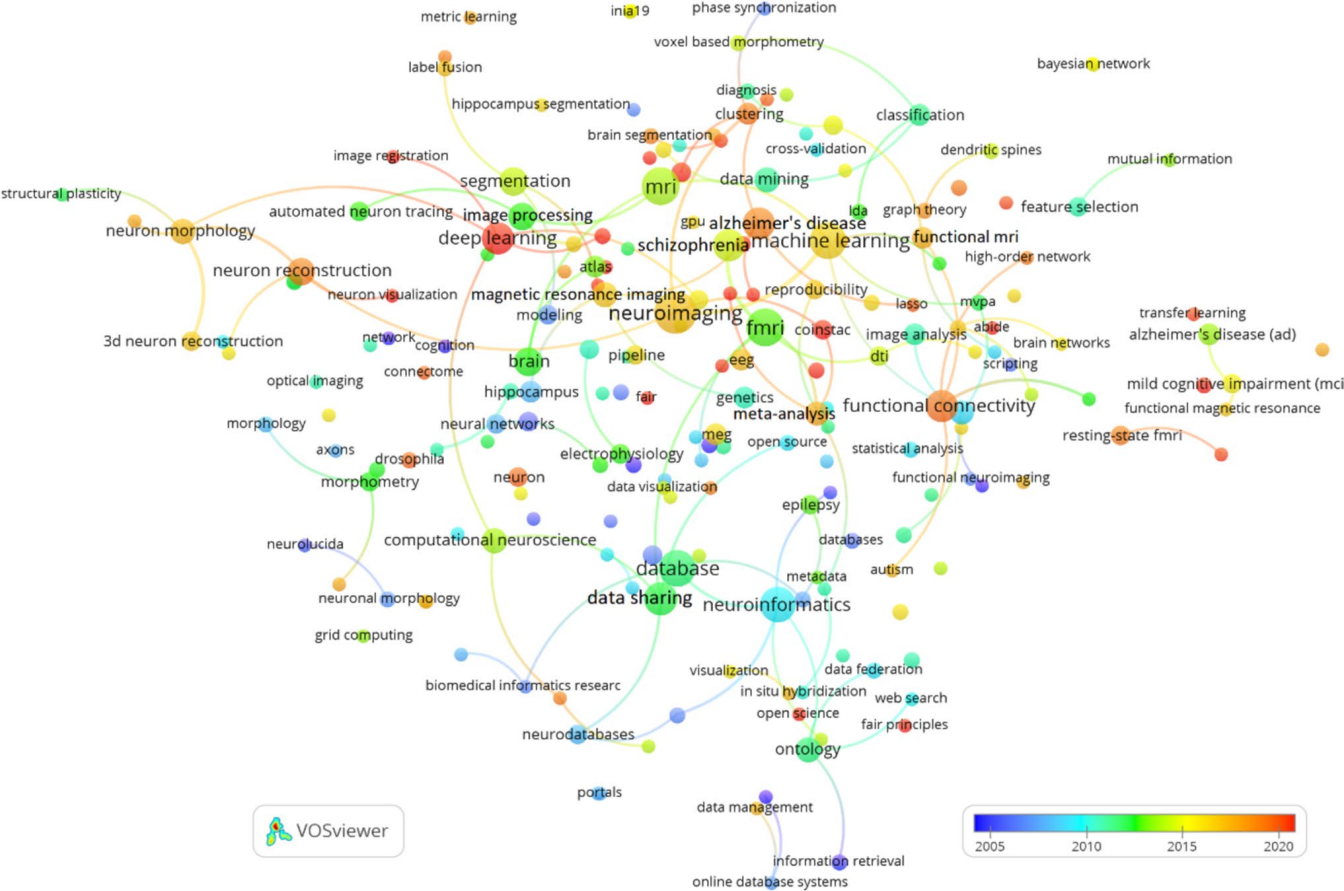
Co-occurrence of author keywords in Neuroinformatics (North America): minimum occurrence threshold of 2 and 100 links

The co-occurrence network of author keywords in Neuroinformatics research from North America is displayed in Figure 16, with a minimum,occurrence threshold of 2 and 100 links. This visualization highlights the most frequently used keywords in publications and how these terms are interconnected, providing insights into the main research themes and emerging trends in the region.

At the centre of the network, neuroimaging dominates as the primary research focus, with strong connections to terms like fMRI, magnetic resonance imaging (MRI), and functional connectivity. This emphasis underscores the significant role that imaging technologies play in understanding brain function and structure in North American Neuroinformatics research.

The integration of machine learning is evident, particularly in its links to neuroimaging, schizophrenia, and Alzheimer's disease. This reflects the growing use of computational techniques to analyse large neuroimaging datasets and study complex neurological disorders. Keywords like deep learning and image processing further demonstrate the adoption of advanced machine learning methods to enhance brain imaging analysis. The terms functional connectivity and brain networks are crucial in the network, especially about fMRI and functional neuroimaging. This area of research focuses on understanding how different brain regions interact and contribute to overall brain function. The connection to psychiatric and neurological conditions like schizophrenia and Alzheimer's disease highlights the importance of this area in disease studies.

Another critical theme in the network is data sharing and database, reflecting the emphasis on open access to large datasets and collaborative research efforts. The presence of these terms, along with Neuroinformatics, underscores the importance of data management and sharing in advancing the field across North America

Research into neuron morphology and neuron reconstruction also plays a prominent role, with terms like 3D neuron reconstruction emphasizing the importance of anatomical studies. These areas focus on mapping and analysing neuron structure to better understand brain function at the cellular level. Additionally, emerging trends in meta-analysis and data mining are highlighted, showing the increasing use of these techniques to synthesize data and identify patterns across multiple studies. This trend reflects a growing interest in leveraging large datasets to draw broader conclusions and gain new insights into brain function and disorders.

**Fig. 17:**
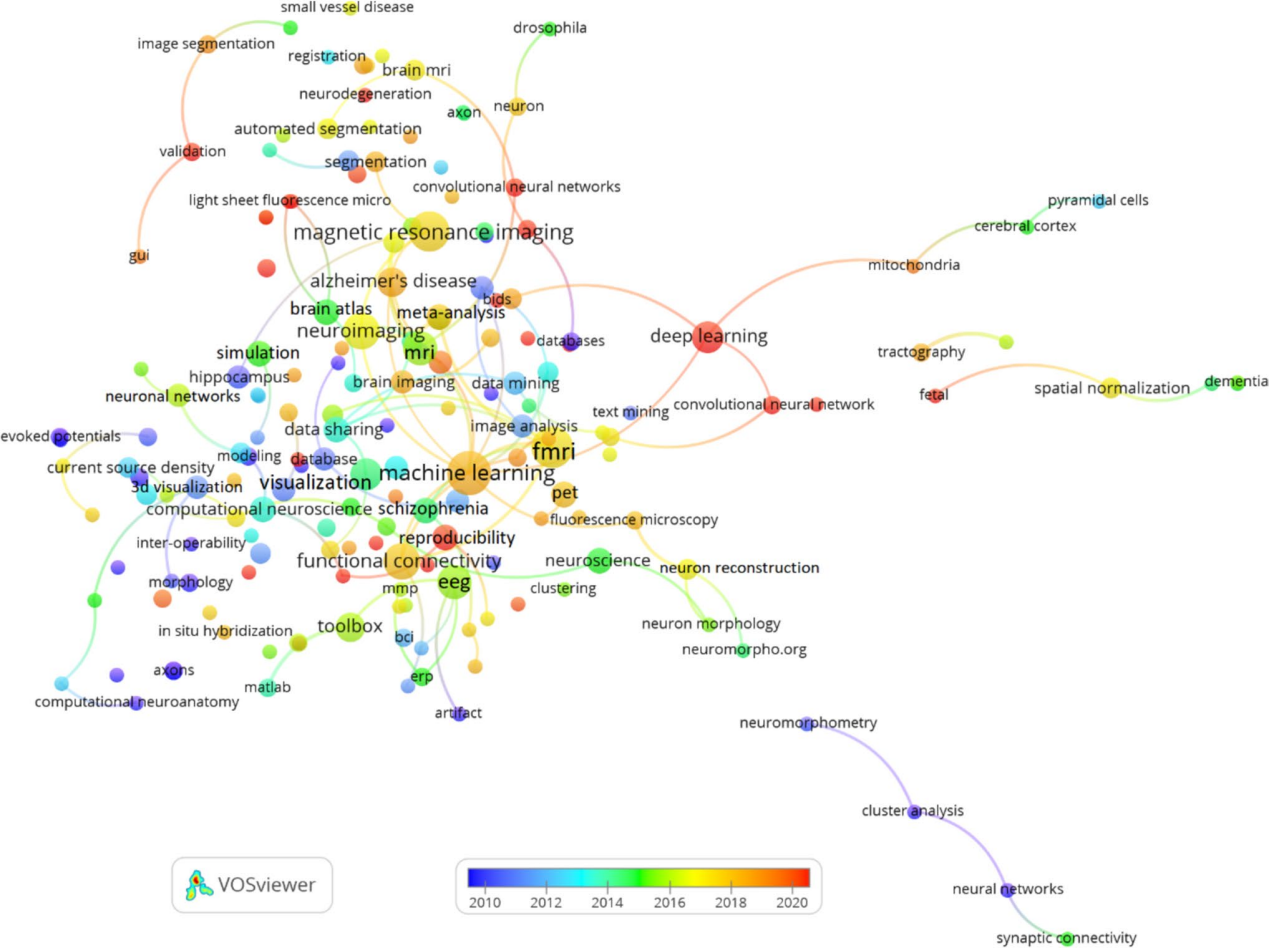
Co-occurrence of author keywords in Neuroinformatics (Europe): minimum occurrence threshold of 2 and 100 links

Figure 17 showcases the co-occurrence network of author keywords in Neuroinformatics research from Europe, with a minimum occurrence threshold of 2 and 100 links. This visualization highlights the most frequently used keywords and how they interrelate, revealing key themes and trends in European Neuroinformatics research.

At the core of the network, magnetic resonance imaging (MRI) and neuroimaging emerge as dominant research focuses, closely linked with other important terms like fMRI, machine learning, and visualization. This indicates a strong emphasis on imaging technologies in the European Neuroinformatics community, with computational methods like machine learning being increasingly applied to analyse and visualize complex neuroimaging data.

Deep learning appears as another significant theme, connected with terms like convolutional neural networks, data mining, and image segmentation, reflecting the growing role of advanced machine learning techniques in the field. These technologies are particularly relevant for analysing large-scale datasets, making them integral to research into brain imaging and neurological diseases.

The network also highlights the importance of Alzheimer's disease and schizophrenia in European research, suggesting a focus on understanding these disorders through neuroimaging and computational analysis. Functional connectivity and reproducibility are key keywords connected to these areas, indicating efforts to understand brain connectivity and ensure the reliability of research findings.

In addition, data sharing and database are central to the network, underscoring the European focus on collaborative research and open access to neuroimaging and Neuroinformatics data. The integration of databases and shared resources is critical for advancing the field and ensuring wide-reaching impacts on neuroscience research.

Keywords such as neuron morphology, neuron reconstruction, and neural networks reflect ongoing research into the structural aspects of neurons and the use of computational tools to map brain connectivity at both the cellular and network levels. This research theme plays a crucial role in expanding our understanding of the brain's anatomy and its functional connections.

**Fig. 18:**
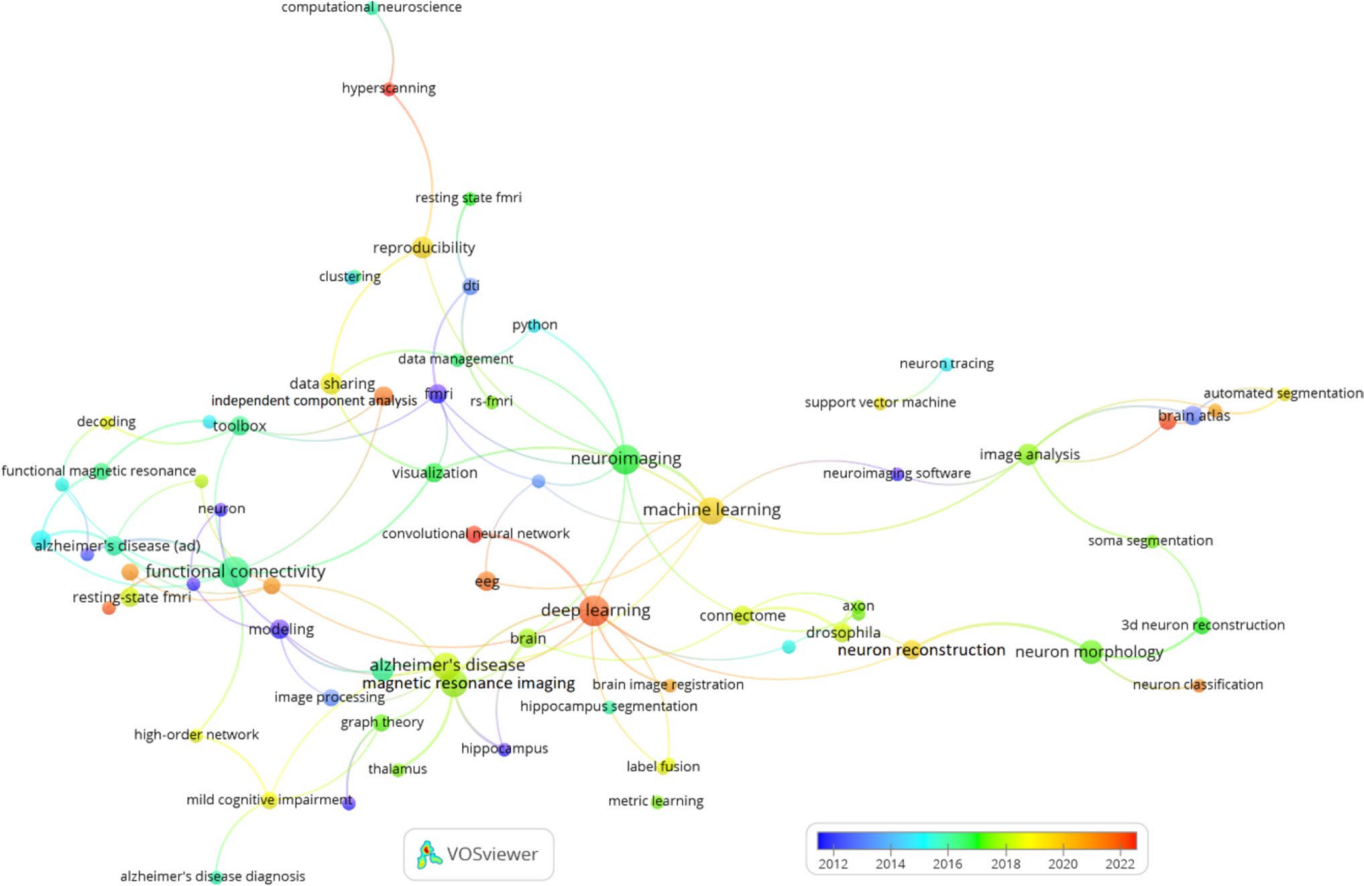
Co-occurrence of author keywords in Neuroinformatics (Rest of the World): minimum occurrence threshold of 2 and 100 links

The co-occurrence network of author keywords in Neuroinformatics research from the "Rest of the World" is illustrated in Figure 18, using a minimum occurrence threshold of 2 and 100 links. This network reveals frequently co-occurring keywords in publications, highlighting the key research areas and emerging trends in these regions.

At the centre of the network, neuroimaging and machine learning emerge as dominant themes, connected to important terms like deep learning, functional connectivity, and magnetic resonance imaging (MRI). These connections underscore the focus on applying machine learning techniques to analyse neuroimaging data. The links to visualization, convolutional neural networks, and support vector machines further emphasize the central role of computational tools in advancing Neuroinformatics research.

The importance of Alzheimer's disease and functional connectivity is also evident, particularly through their association with resting-state fMRI and functional magnetic resonance imaging. This shows a significant emphasis on studying neurological conditions and understanding brain connectivity through advanced imaging techniques. Additionally, there is a strong focus on neuron morphology and neuron reconstruction, with keywords like 3D neuron reconstruction and axon tracing connected to these themes. This cluster reflects an interest in exploring the structural characteristics of neurons and reconstructing their forms for detailed analysis.

Key themes of data sharing and reproducibility are also present in the network, indicating a growing emphasis on collaborative research and ensuring the reliability of findings across studies. Keywords such as data management and toolbox highlight the development of computational tools to facilitate these collaborative efforts. Other important terms include image analysis, brain atlas, and automated segmentation, which emphasize the use of computational methods for processing and segmenting brain imaging data. The strong connection to image processing and segmentation underscores the technological advancements driving Neuroinformatics.

Table 13 provides a global and temporal analysis of the co-occurrence of author keywords in Neuroinformatics, highlighting the most frequently occurring keywords across different time periods. The analysis reveals how research focus has evolved from 2003 to 2023, reflecting shifts in methodologies, technologies, and areas of interest.

Globally, Machine Learning and Neuroimaging are the most dominant keywords, each appearing 32 times, indicating their central role in Neuroinformatics research. FMRI and Magnetic Resonance Imaging (MRI), both with 27 occurrences, also stand out, underlining the importance of imaging techniques in understanding brain function. Functional Connectivity (24 occurrences) and Deep Learning (23 occurrences) further demonstrate the growing reliance on computational methods to analyse complex neural data. Over time, Data Sharing (18 occurrences) and EEG (17 occurrences) have also become key topics, indicating a trend toward openness in research and increased use of neurophysiological data.

The 2019–2023 period is characterized by the prominence of Deep Learning (21 occurrences) and Machine Learning (18 occurrences), signalling the increasing application of artificial intelligence techniques in neuroimaging and brain data analysis. Functional Connectivity (15 occurrences) and Alzheimer’s Disease (13 occurrences) reflect the focus on understanding brain networks and neurodegenerative diseases in recent years. The rise of Convolutional Neural Networks (12 occurrences) and Reproducibility (7 occurrences) emphasizes the growing concern for both advanced computational tools and research reliability.

In the 2014–2018 timeframe, Magnetic Resonance Imaging (MRI) and Neuroimaging were leading terms with 9 occurrences each, highlighting the focus on imaging technologies. Alzheimer's Disease (7 occurrences) and FMRI (5 occurrences) also maintained importance, pointing to ongoing efforts to explore neurodegenerative disorders and brain function. During this period, research expanded into areas such as Neuron Morphology and 3D Neuron Reconstruction (5 occurrences each), indicating an interest in the structural aspects of brain cells.

In the earlier period from 2003–2013, Database (13 occurrences) and FMRI (12 occurrences) were leading keywords, reflecting the foundational focus on data management and imaging techniques. Neuroinformatics (11 occurrences) itself emerged as a central theme, alongside terms like Data Sharing (10 occurrences) and MRI (9 occurrences), highlighting early efforts to build collaborative infrastructures and integrate various neuroimaging modalities. Keywords like Ontology and Brain Atlas (8 and 7 occurrences, respectively) underscore the initial focus on building frameworks for organizing brain data.

**Table 13:** Co-occurrence of author keywords in Neuroinformatics: global and temporal analysis

	Global		2019–2023		2014–2018		2003–2013	
R	Keyword	Occ	Keyword	Occ	Keyword	Occ	Keyword	Occ
1	Machine Learning	32	Deep Learning	21	Machine Learning	9	Database	13
2	Neuroimaging	32	Machine Learning	18	Magnetic Resonance Imaging	9	FMRI	12
3	FMRI	27	Neuroimaging	16	MRI	9	Neuroinformatics	11
4	Magnetic Resonance Imaging	27	Functional Connectivity	15	Alzheimer’s Disease	7	Data Sharing	10
5	MRI	26	Alzheimer’s Disease	13	Neuroimaging	6	MRI	9
6	Functional Connectivity	24	Magnetic Resonance Imaging	13	Computational Model	5	Neuroimaging	9
7	Deep Learning	23	Convolutional Neural Networks	12	FMRI	5	Ontology	8
8	Alzheimer’s Disease	22	FMRI	10	Neuron Morphology	5	Brain Atlas	7
9	Data Sharing	18	EEG	9	Neuron Reconstruction	5	Hippocampus	7
10	Database	17	MRI	7	3D Neuron Reconstruction	4	Modeling	7
11	EEG	17	Reproducibility	7	Data Mining	4	Computational Neuroscience	6
12	Neuroinformatics	17	Meta-Analysis	6	Drosophila	4	EEG	6
13	Segmentation	16	Neuron Reconstruction	6	Feature Selection	4	Functional Connectivity	6
14	Schizophrenia	14	Schizophrenia	6	GPU	4	Morphology	6
15	Reproducibility	13	Bids	5	Image Processing	4	Segmentation	6
16	Brain	12	Clustering	5	Neuroinformatics	4	Simulation	6
17	Computational Neuroscience	12	Data Sharing	5	Segmentation	4	3D Visualization	5
18	Convolutional Neural Networks	12	Independent Component Analysis	5	Automated Segmentation	3	Brain	5
19	Brain Atlas	11	Segmentation	5	Axon	3	Connectivity	5
20	Neuron Reconstruction	11	Brain	4	Brain	3	Data Mining	5
21	Ontology	11	Brain Imaging	4	Brain MRI	3	Neuroanatomy	5
22	Visualization	11	Classification	4	Classification	3	Neuroimaging Software	5
23	Classification	10	COINSTAC	4	Data Sharing	3	Schizophrenia	5
24	Data Mining	10	Computational Neuroscience	4	Dendrite	3	Visualization	5
25	Image Analysis	10	Connectome	4	Diffusion Tensor Imaging	3	Data Federation	4
26	Image Processing	10	Diffusion MRI	4	Functional Connectivity	3	Data Integration	4
27	Image Segmentation	10	Functional MRI	4	Image Analysis	3	Databases	4
28	Meta-Analysis	10	Image Segmentation	4	Image Registration	3	Epilepsy	4
29	Toolbox	10	Neuron	4	Neuron	3	Gene Expression	4
30	Feature Selection	9	Resting-State FMRI	4	Neuronal Networks	3	Image Analysis	4

*Occ *= Occurrences

The leading topics in Neuroinformatics research between 2013 and 2022 are detailed in Table 14, which is based on data from Scopus and highlights key areas of focus along with their field-weighted citation impact (FWCI) and worldwide prominence percentile (PP). The topics and topic clusters are available in Scopus through the SciVal platform, providing further insights into the research landscape (SciVal, 2024). This analysis underscores the topics that have garnered the most attention and impact in the field over the past decade. In case of the same number of publications, the percentile of global prominence will be considered (Klavans and Boyack 2017). Considering that a publication can only belong to one topic and group.

The highest-ranked topic, Neurite, Axon, and 3D Imaging, led the list with 48 total papers and an impressive FWCI of 2.14, placing it in the 81.253 percentile for global impact. This indicates a strong emphasis on understanding the detailed structures of neurons and their connections through advanced imaging techniques (Purkayastha et al., 2019).

Another prominent topic, Magnetic Resonance Imaging (MRI), Functional Connectivity, and Brain Mapping, appears with 19 papers and an FWCI of 2.94, which positions it in the 99.496 percentile, making it one of the most influential research areas in terms of citation impact.

Research combining Diffusion MRI, Image Processing, and Diffusion Tensor Imaging also plays a significant role, although with a lower FWCI of 0.54, it reflects an area of active exploration despite not achieving as high a citation impact as other topics. Meanwhile, studies on Alzheimer's Disease, MRI, and Neurodegenerative Disorders reflect a critical focus on neurological diseases, with 11 papers and a notable FWCI of 2.85, placing the research in the 99.279 percentile, highlighting its global prominence.

Other notable topics include Data Sharing, MRI, and Information Dissemination with an FWCI of 0.56, which, although lower, emphasizes the importance of making research findings widely accessible. Functional Connectivity, MRI, and Brain Mapping has also garnered attention, with an FWCI of 1.04, positioning this area of study in the 97.405 percentile. In terms of technological advances, research focusing on Electron Microscopy, Synapse, and Focused Ion Beam and Magnetoencephalography, Electrophysiology, and Brain Mapping indicates important developments in high-resolution brain imaging and electrophysiological analysis, with respective FWCIs of 1.8 and 0.48.

**Table 14:** Leading topics in neuroinformatics between 2013 and 2022 (Scopus)

*R*	Topic	TP	FWCI	PP
1	Neurite; Axon; 3D Imaging	48	2.14	81.25
2	Magnetic Resonance Imaging; Functional Connectivity; Brain Mapping	19	2.94	99.49
3	Diffusion MRI; Image Processing; Diffusion Tensor Imaging	13	0.54	96.18
4	Magnetic Resonance Imaging; Image Processing; Hippocampus	13	1.03	89.55
5	Magnetic Resonance Imaging; Neural Network; Brain Mapping	12	1.53	96.39
6	Alzheimer’s Disease; Magnetic Resonance Imaging; Neurodegenerative Disorder	11	2.85	99.27
7	Data Sharing; Magnetic Resonance Imaging; Information Dissemination	11	0.56	57.03
8	Functional Connectivity; Magnetic Resonance Imaging; Brain Mapping	10	1.04	97.40
9	Connectomics; Neuroscience; Neural Pathway	10	1.12	40.59
10	Neural Network; Neuroscience; Computer Simulation	9	2.13	63.64
11	Magnetoencephalography; Electrophysiology; Brain Mapping	7	0.48	92.59
12	Electron Microscopy; Synapse; Focused Ion Beam	6	1.8	92.19
13	Medical Imaging; Computer Assisted Tomography; Image Registration	5	1.23	94.87
14	Magnetic Resonance Imaging; Image Processing; Cerebral Cortex	5	1.08	88.93
15	Magnetic Resonance Imaging; Dementia Praecox; Brain Mapping	5	1.75	78.94
16	Electrophysiology; Reproducibility; Data Type	5	2.85	68.42
17	Magnetic Resonance Imaging; Skull; Image Segmentation	5	0.59	67.84
18	Magnetic Resonance Imaging; Anterior Commissure; Image Processing	5	0.97	41.44
19	Electroencephalography; Computer Interface; Biomedical Signal Processing	4	1.32	99.45
20	Imaging Genetics; Single-Nucleotide Polymorphism; Magnetic Resonance Imaging	4	0.36	76.11
21	Magnetic Resonance Imaging; DNA Template; Brain Mapping	4	0.37	65.57
22	Gene Expression; Transcriptome; Neuroanatomy	4	0.39	21.98
-	13 Topics	3	-	-
-	21 Topics	2	-	-
-	121 Topics	1	-	-

*R *= Rank; *TP* = Total papers; *FWCI* = Field-weighted citation impact (data from Scopus); *PP* = Worldwide prominent percentile (according to Scopus and FWCI)

Table 15 presents the leading topic clusters in Neuroinformatics research from 2013 to 2022, according to Scopus data. The top cluster, "Functional Magnetic Resonance Imaging (FMRI); Brain Mapping; Electroencephalography," consists of 144 papers, with a field-weighted citation impact (FWCI) of 1.4 and a global prominence percentile (PP) of 91.367. This indicates a strong focus on neuroimaging and brain activityresearch within the field.

Other significant clusters include "Homeodomain Protein; Behaviour (Neuroscience); RNA Interference," which has 49 papers and an FWCI of 2.1, focusing on genetic and behavioural aspects of neuroscience. Another important cluster is "Brain-Computer Interface; Electroencephalography; Biomedical Signal Processing," highlighting advancements in neurotechnology and human-computer interactions.

The table further details clusters related to imaging techniques, such as "Magnetic Resonance Imaging; Tau; Cognitive Function" and "Image Segmentation; Deep Neural Network; Object Detection," showcasing the integration of machine learning and deep neural networks in image analysis. These clusters emphasize the interdisciplinary nature of Neuroinformatics, spanning areas like imaging, genetics, brain functionality, and computational modeling.

**Table 15:** Leading topic clusters in neuroinformatics between 2013 and 2022 (Scopus)

*R*	Topic Cluster	TP	FWCI	PP
1	Functional Magnetic Resonance Imaging; Brain Mapping; Electroencephalography	144	1.4	91.36
2	Homeodomain Protein; Behavior (Neuroscience); RNA Interference	49	2.1	36.10
3	Brain-Computer Interface; Electroencephalography; Biomedical Signal Processing	21	1.18	85.54
4	Magnetic Resonance Imaging; Tau; Cognitive Function	20	2.26	91.30
5	Gaussian Distribution; Signal-to-Noise Ratio; Photonics	16	1.57	91.04
6	Transcranial Magnetic Stimulation; Motor Cortex; Diffusion Tensor Imaging	16	0.53	77.50
7	Image Segmentation; Deep Neural Network; Object Detection	15	2.82	99.93
8	Magnetic Resonance Imaging; Relapsing Remitting Multiple Sclerosis; Quality of Life	10	0.83	90.58
9	Visual Cortex; Motion Perception; Calcium Imaging	8	0.47	38.91
10	Gene Expression Profiling; RNA Sequencing; Ontology	6	0.54	92.54
11	Empathy; Visual Perception; Functional Magnetic Resonance Imaging	6	0.6	80.51
12	Oligodendrocyte; Central Nervous System; Hippocampus	5	2.64	80.57
13	Electroencephalography in Epilepsy; Temporal Lobe; Hippocampus	4	0.86	70.56
14	Gaussian Distribution; Regression Analysis; Model Selection	4	0.36	43.95
15	Gaussian Distribution; Optical Vortex; Fluorescence Microscopy	3	1.44	77.10
16	Hippocampus; Neurotransmission; Neuronal Plasticity	3	0.35	71.15
17	Cerebellum; Eye Movement; Purkinje Cell	3	0.4	55.65
18	Time Series Analysis; Electroencephalography; Information Theory	3	1.61	19.29
-	16 Topic Clusters	2	-	-
-	49 Topic Clusters	1	-	-

Abbreviations are available in Table 14

## Conclusions

The bibliometric analysis of Neuroinformatics over the past 20 years provides key insights into its evolution as a multidisciplinary journal at the intersection of neuroscience, computational science, and data-driven research. Our study highlights the significant trends in publication and citation activity, showing a consistent rise in both areas, particularly in the last decade as computational neuroscience has gained substantial momentum. The journal’s early years were marked by steady growth, but from 2013 onwards, there has been a remarkable increase in the number of published papers, peaking notably in 2022 with a record number of publications. This surge is indicative of growing interest in Neuroinformatics as technological advancements, such as machine learning, artificial intelligence, and big data analytics, drive the need for sophisticated computational tools in brain science research.

The dominant research themes, such as neuroimaging, data sharing, machine learning, and functional connectivity, emphasize the journal’s role in addressing critical challenges within neuroscience using computational methodologies. Neuroimaging has consistently remained at the forefront, with advancements in magnetic resonance imaging (MRI) and functional MRI (fMRI) forming the backbone of much of the research published. In parallel, the growing focus on data sharing and open science practices has promoted collaborative research efforts that foster transparency and reproducibility—core values that are increasingly essential in scientific progress. Furthermore, emerging topics like deep learning, neuron reconstruction, and reproducibility demonstrate the field’s adaptability to new technologies and research paradigms. These innovations enable researchers to approach brain science from new angles, facilitating deeper insights into brain function, structure, and disorders.

Bibliometric analyses, such as co-citation and bibliographic coupling, reveal Neuroinformatics' deep connections with other leading journals in neuroscience and computational research. This highlights the journal’s pivotal role in advancing both computational methods and neurobiological research. The journal's influence spans multiple scientific disciplines, acting as a bridge between neuroscience, bioinformatics, and data science. This interdisciplinary approach has attracted a broad spectrum of contributors, as seen through the global map of top authors, institutions, and countries driving innovation in the field. The USA, China, and Europe remain the most prolific regions, withresearchers from these areas significantly contributing to the journal's intellectual output and research impact. Notably, the USA and China have seen a surge in Neuroinformatics research in recent years, likely driven by their investments in advanced computational infrastructures and large-scale brain research initiatives.

The collaboration patterns revealed in co-authorship and keyword cooccurrence analyses further highlight the journal’s interdisciplinary and collaborative nature. Neuroinformatics serves as a platform where experts from diverse fields—including neuroscience, computer science, mathematics, and bioengineering—come together to address the complex challenges posed by brain research. This cross-disciplinary collaboration is reflected in the increasing complexity of research topics, which now frequently involve advanced computational models, big data analysis, and high-performance computing. These collaborations have not only fostered the exchange of ideas but have also promoted the development of new methodologies and innovative approaches to solving neuroscience problems, for example, the growth of foundational generative artificial intelligence modeling (GAIM) (DuPre and Poldrack, 2024).

Looking to the future, Neuroinformatics is well-positioned to continue influencing the trajectory of brain research as computational technologies and neuroscience further converge. The persistent and emerging themes identified in this bibliometric analysis suggest that the journal will remain at the forefront of scientific innovation. With the rise of AI-driven research, neuroimaging and fMRI techniques becoming more sophisticated, and global research collaborations continuing to expand, the journal is expected to play a critical role in shaping the next generation of Neuroinformatics research (Abrams and Van Horn, 2024). Additionally, the journal’s increasing citation impact highlights its growing recognition as a key resource for cutting-edge research, underscoring its importance in guiding future developments (Geminiani et al., 2024; Rokem and Benson, 2024).

This comprehensive bibliometric study of Neuroinformatics not only celebrates the journal’s significant contributions over the past two decades but also provides a roadmap for future research directions. The journal has successfully adapted to the evolving landscape of neuroscience and computational modeling, ensuring its continued relevance in a rapidly advancing field. As both persistent and emerging research themes are explored, the journal will remain a vital resource for scholars and practitioners alike, contributing to the development of innovative solutions for understanding brain function and neurological disorders. Ultimately, Neuroinformatics is poised to lead in advancing knowledge and fostering collaboration at the crossroads of neuroscience and computational science, ensuring its position as a key player in the global research community.

## Data Availability

No datasets were generated or analysed during the current study.
